# Comparative Venomics of *C. flavidus* and *C. frigidus* and Closely Related Vermivorous Cone Snails

**DOI:** 10.3390/md20030209

**Published:** 2022-03-15

**Authors:** S. W. A. Himaya, Alexander Arkhipov, Wai Ying Yum, Richard J. Lewis

**Affiliations:** Institute for Molecular Bioscience, The University of Queensland, St Lucia, Brisbane, QLD 4072, Australia; alexander.arkhipov@uq.net.au (A.A.); wai.yum1@uq.net.au (W.Y.Y.)

**Keywords:** conotoxin, cone snails, venom diversity, proteomics, transcriptomics

## Abstract

Cone snail venom biodiversity reflects dietary preference and predatory and defensive envenomation strategies across the ≈900 species of *Conidae*. To better understand the mechanisms of adaptive radiations in closely related species, we investigated the venom of two phylogenetically and spatially related species, *C. flavidus* and *C. frigidus* of the *Virgiconus* clade. Transcriptomic analysis revealed that the major superfamily profiles were conserved between the two species, including 68 shared conotoxin transcripts. These shared transcripts contributed 90% of the conotoxin expression in *C. frigidus* and only 49% in *C. flavidus*, which showed greater toxin diversification in the dominant O1, I2, A, O2, O3, and M superfamilies compared to *C. frigidus*. On the basis of morphology, two additional sub-groups closely resembling *C. flavidus* were also identified from One Tree Island Reef. Despite the morphological resemblance, the venom duct proteomes of these cryptic sub-groups were distinct from *C. flavidus.* We suggest rapid conotoxin sequence divergence may have facilitated adaptive radiation and the establishment of new species and the regulatory mechanisms facilitating species-specific venom evolution.

## 1. Introduction

Cone snails (Caenogastropoda: Conidae) are a relatively recent lineage of ≈900 species of marine molluscs [1], with origins dating back 55–73 million years (mya) [2]. They are typically found in tropical and subtropical waters [3], mostly distributed around coral reefs in the Indo-Pacific region [4,5]. Despite their relatively recent origins, Conidae have extraordinary taxonomic and ecological diversity [6,7], with the fastest diversification rate among gastropods [8]. Although recent advances in molecular biology and next generation sequencing have helped dissect their phylogeny [6,7,9,10,11,12], our understanding of the role venom peptide biodiversity plays in speciation remains incomplete.

All cone snails are specialised predators that utilise a complex cocktail of venom peptides for defence and to facilitate predation on worms, other molluscs, and small fish. These specialised venoms are produced along a compartmentalised venom duct and delivered by injection through a hollow radula tooth [13]. On the basis of the venom composition of over 200 species of cone snails, it appears that each species produces a unique venom reflecting their ancestry with adaptations to exploit different ecological niches [3,6,7,10,14]. 

Cone snail species are typically distinguished by morphological features including shell shape, shell pattern, foot muscle pattern, and syphon colour. However, cryptic species complexes such as the *C. sponsalis* species complex [15] and *C. flavidus/C. frigidus* species complex [16] are more challenging to separate using morphological characteristics alone. Despite multiple efforts to understand the molecular phylogeny and evolutionary history within different clades of cone snails [15,16,17], changes in venom composition within closely related species has not been extensively studied [18,19]. Given their phylogenetic relatedness, we expected that studying the *C. flavidus/C. frigidus* cryptic species complex would help understand how venom diverges during speciation within a clade.

This study investigated the expression and diversification of conotoxins in closely related *C. flavidus/C. frigidus* species complex of the *Virgiconus* clade using a venomics approach. Specimens sharing the same habitat was used for this comparative study to avoid environmental variations. The conotoxin profiles of the venom duct transcriptomes and proteomes were compared and contrasted between the two specimens. The venom compositions of these closely related *Virgiconus* species provide unique evidence to the divergence of venom components during speciation as well as adaptive ability of the individual species that may have diverged from a common ancestor.

## 2. Results

### 2.1. Comparative Conotoxin Profiles of C. flavidus and C. frigidus

**Figure 1:**
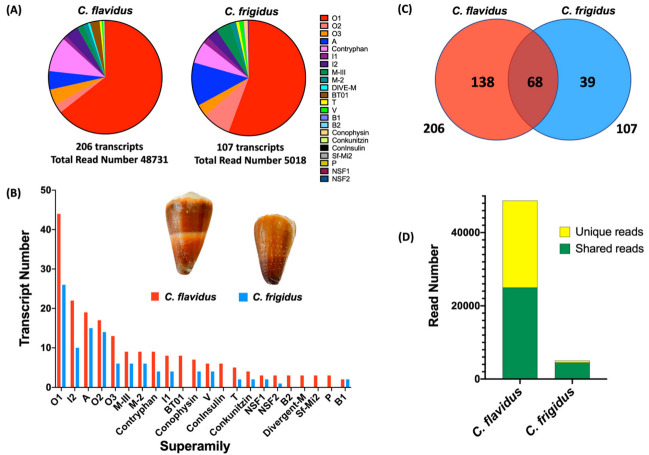
Distribution of conotoxins in *C. flavidus* and *C frigidus* venom duct transcriptomes. (**A**) Expression levels (relative read number) of venom peptide superfamilies in *C. flavidus* and *C. frigidus*. (**B**) Number of transcripts in each superfamily found in the venom duct transcriptomes of *C. flavidus* and *C. frigidus*. (**C**) Venn diagram showing precursor overlap between the conotoxin transcripts identified in *C. flavidus* and *C. frigidus* venom ducts. (**D**) Expression levels (read numbers) of unique precursors and shared precursors in the *C. flavidus* and *C. frigidus* venom duct transcriptomes.

Comparative analysis of the venom duct transcriptomes of *C. flavidus* and *C. frigidus* revealed a total of 245 conotoxin precursors, including 68 shared between the two species. *C. flavidus* expressed 206 conotoxin precursor sequences classified into 21 gene superfamilies, while *C. frigidus* expressed 107 conotoxin precursor sequences from 17 gene superfamilies (Figure 1, Table 1 and Table S1). This level of similarity is reminiscent of the overlap observed within the venoms of individuals from the same species [20,21,22,23], confirming *C. flavidus* and *C. frigidus* are indeed closely related. Surprisingly, the conotoxin sequence number was ≈2 fold higher, and the total read number ≈9.5 fold higher in the *C. flavidus* venom duct transcriptome compared to that of *C. frigidus*, although similarities in the relative distribution of these transcripts across the superfamilies was maintained (Figure 1B, Table 1).

For both species, superfamilies O1, I2, A, O2, O3, M, and contryphans dominated, contributing 133 conotoxin precursors (92.5% of the total conotoxin expression) in *C. flavidus* and 81 conotoxin precursors (92.3% of the total conotoxin expression) in *C. frigidus*. Except for the minor superfamilies B2, bt-01, Divergent M-, sf-mi2, P, and Con-Insulin, all other superfamilies were found in both species at a similar ratio of relative expression and transcript numbers (Figure 1A,B, Table 1). Two new superfamilies of conotoxins found in both *C. flavidus* and *C. frigidus* transcriptomes were named NSF1 and NSF2. Three precursors of NSF1 and three precursors of NSF2 were found in *C. flavidus*, while *C. frigidus* had one NSF1 precursor and one NSF2 precursor (Figure S1) that were also found in *C. flavidus*. The mature sequence of both these superfamilies were cysteine rich, with NSF1 superfamily having framework VI/VII [23,24] and NSF2 in the recently described XXVII framework [25].

The 68 conotoxin transcripts common to both species belonged to 14 superfamilies, with most (59) from the dominant superfamilies (Figure S2, Table 1). Interestingly, these common transcripts contributed 48.9% of *C. flavidus* and 89.7% of *C. frigidus* total conotoxin reads (Figure 1D). Although 39 conotoxin precursors were found exclusively in *C. frigidus*, their expression level (in read numbers) was limited (10.5%) (Figure 1D), indicating that the majority of its dominant conotoxins are not species-specific. In contrast, a relatively higher number of conotoxins were unique to *C. flavidus* (135 of 206) accounted for 51% of total conotoxin read level (Figure 1D). Among these 135 unique conotoxins in *C. flavidus*, only 26 transcripts (2.4% of the total reads) belonged to minor superfamilies only present in *C. flavidus*. 

#### Comparative Proteomic Search of the Trasncriptomic Sequences in Respective Venom Duct Proteomes

**Figure 2:**
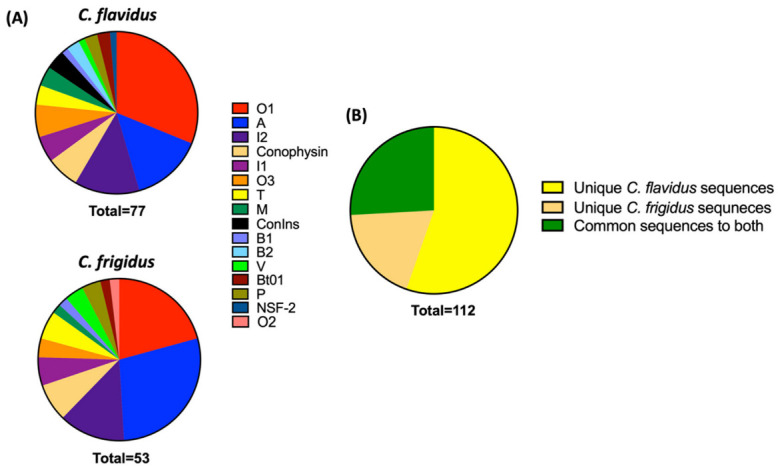
Proteomic complexity of *C. flavidus* and *C. frigidus*. (**A**) Superfamily distribution of identified sequences in *C. flavidus* and *C. frigidus* proteomes using ProteinPilot software. (**B**) Distribution of unique and common sequences identified in *C. flavidus* and *C. frigidus* proteomes.

To identify which transcripts were translated into conotoxins in the venom duct, transcriptomic sequences were searched against reduced, alkylated, and trypsin-digested whole venom duct extracts of *C. flavidus* and *C. frigidus* using a sequence integration algorithm in ProteinPilot. This preliminary proteomic search predicted 50 full length and 62 partial conotoxin sequences with 99% confidence (Table S2) distributed across 16 superfamilies (Figure 2A). Sixty two of the 138 unique *C. flavidus* conotoxins, 21 of the 39 unique *C. frigidus* conotoxin sequences, and 29 of the 68 common conotoxin sequences were predicted to be present in the proteome (Figure 2B). Similar to the transcriptomes, O1, A, and I2 conotoxins dominated the proteomes of *C. flavidus* and *C. frigidus*, although sequences from minor superfamilies T, V, I1, O2, O3, B1, B2, NSF1, and NSF2 were also predicted. 

As the major superfamily in both species, sequence information for 32 of 53 O1 conotoxins were detected in the ProteinPilot search of the venom duct proteomes. Twenty-three of these detected sequences showed higher expression levels in their respective transcriptomes (>10 reads). Among the 18 detected I2 sequences, 14 are highly expressed in the respective transcriptomes and all four of the common I2 conotoxins to both species were detected through this proteome search. Nineteen A superfamily conotoxins belonging to all three structural groups (4/7, 4/6, and 4/5) were found in the ProteinPilot search. Overall, sequence evidence of 11 unique *C. flavidus* transcripts were also found in the venom duct proteome of *C. frigidus*, and 02 unique *C. frigidus* transcripts were also found in the venom duct proteome of *C. flavidus*, which were not shared between the two species at the transcriptome level (Table S2). This disparity could have been due to the variability in toxin expression within individuals of the same species as the proteome and transcriptomes were performed on the venom ducts of different individuals. 

### 2.2. Conotoxin Diversity in O1, I2, A and Contryphan Superfamilies of C. flavidus and C. frigidus at the Transcriptomic Level

**Figure 3:**
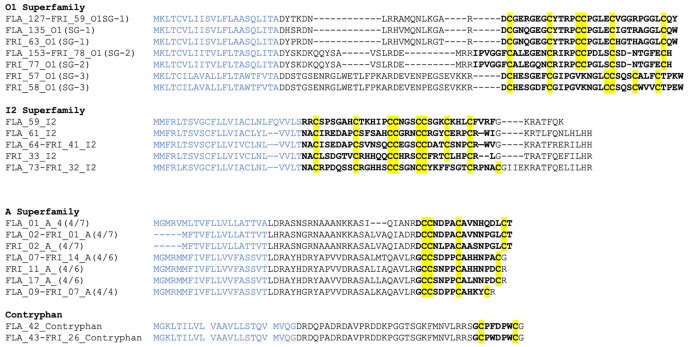
Representative precursors from the major superfamilies O1, I2, A, and contryphan found in *C. flavidus* and *C. frigidus* venom duct transcriptomes. The signal sequence is indicated in blue colour, and the mature sequence is indicated in bold letters. Cysteines are highlighted in yellow. Specific sub-groups within a superfamily are shown in parenthesis after the sequence ID. SG-1, sub-group 1; SG-2, sub-group 2; SG-3, sub-group 3. 4/7, 4/7 cysteine canonical structure in the mature sequence; 4/6, 4/6 cysteine canonical structure in the mature sequence; 4/4, 4/4 cysteine canonical structure in the mature sequence.

O1 was the most abundant conotoxin superfamily in both *C. flavidus* and *C. frigidus* transcriptomes. On the basis of the amino acid sequence of 53 O1 precursors, three sub-groups were identified (Figure 3 and Figure S3). Sub-group 1 comprised 31 closely related O1 precursors (≈80% similarity) contributing ≈90% of total O1 superfamily expression in both species. Interestingly, the most abundant conotoxin transcript in *C. flavidus* (FLA_127_O1; 7076 reads) and *C. frigidus* (FRI_59_O1; 1102 reads) were identical. O1 sub-group 2 comprised 22 sequences that had pro-peptide and mature sequences distinct from sub-group 1. These sequences showed ≈62% similarly and accounted for only 12% of total O1 expression in *C. flavidus* and 10% in *C. frigidus*. The third sub-group contained only 2 *C. frigidus* derived O1 precursors (FRI_57 and FRI_58), which were the most divergent at both pro-and mature sequence levels, with ≈30% sequence identity O1 sub-group 1 and ≈35% sequence identity to O1 sub-group 2. 

I2 was the next major superfamily in both species, with 22 precursors in *C. flavidus* and 10 precursors in *C. frigidus* (3.9 and 3.1% of total conotoxin reads, respectively). All I2 superfamily precursors share the typical XI cysteine framework (Figure 3 and Figure S4). Among the 28 unique I2 precursors, only four were common to both species, and unlike O1, the most abundant precursors FLA62 and FRI33 were not identical, showing relatively high sequence diversity in I2 superfamily between the two species.

Twenty-five unique A superfamily transcripts were also identified in the venom ducts of *C. flavidus* (19 precursors accounting for 5.2% of total reads) and *C. frigidus* (15 precursors accounting for 12.7% of total reads), with 08 common to both transcriptomes (Figure S5). All A superfamily conotoxins had framework I cysteine frameworks 4/7, 4/6, or 4/5 (Figure 3). The 4/6 cysteine framework dominated, with 17 unique precursors including the commonly expressed FLA_07-FRI_14 that contributed 70% and 81% of total A conotoxin expression in *C. flavidus* and *C. frigidus*, respectively. The two 4/5 framework conotoxins also showed relatively high read numbers in *C. flavidus* (22%) and *C. frigidus* (11.6%), while the 4/7 framework conotoxins were less abundant in both *C. flavidus* (7.8%) and *C. frigidus* (7.3%). 

Fewer sequence number of contryphans were found in *C. flavidus* (9) and *C. frigidus* (4) (Figure 3 and Figure S6). Despite the low precursor numbers, these contriphans accounted for 10% and 6% of total read numbers in *C. flavidus* and *C. frigidus*, respectively. Two contryphans with high read numbers were common to both the *C. flavidus* and *C. frigidus* transcriptomes (FLA_43_Contry and FRI_31_Contry). 

### 2.3. Peptide Mass Expression Patterns in the C. flavidus and C. frigidus Venom Duct Sections

**Figure 4:**
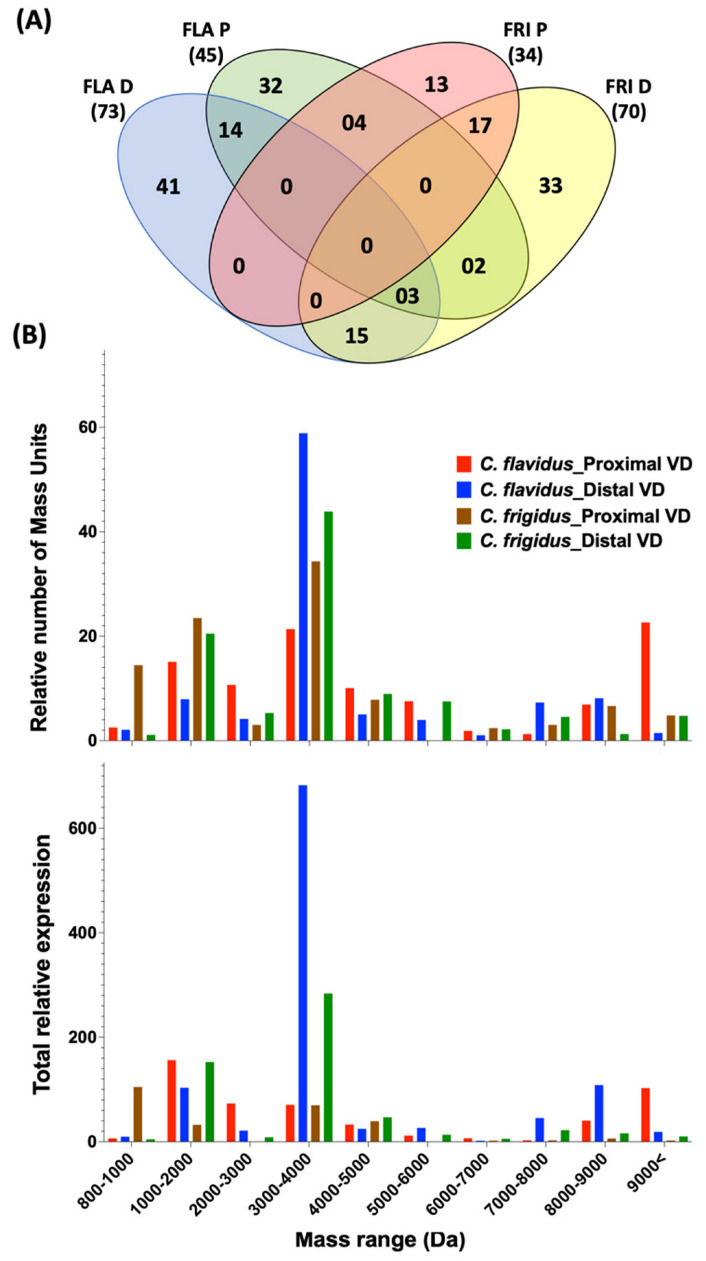
Peptide mass distribution across proximal and distal venom ducts of *C. flavidus* and *C. frigidus.* (**A**) Four-way Venn diagram showing overlap in masses (>1% relative intensity), with the total number of masses considered for the analysis shown in parentheses. The full mass list and relative abundance for each peptide mass unit in each duct section are shown in Table S3. (**B**) The relative number of mass units (top panel) and the sum of their relative expression (bottom panel) for masses ranging from 800 – 9000 Da. FLA D—*C. flavidus* distal venom duct; FLA P—*C. flavidus* proximal venom duct; FRI D—*C. frigidus* distal venom duct; FRI P—*C. frigidus* proximal venom duct.

The expression levels and the overlap of the 174 dominant peptide masses (relative intensities >1%) identified from the proximal and distal venom duct sections of *C. flavidus* and *C. frigidus* (Figures S7–S10, Table S3) were compared, and the overlap is shown in a Venn diagram (Figure 4A). Most overlap was observed between the distal and proximal venom duct sections of each species, *C. flavidus* (14) and *C. frigidus* (17). Fifteen masses were commonly found in the distal sections of *C. flavidus* and *C. frigidus* venom ducts, and four masses were common to proximal sections. Interestingly, no masses were common to all four tissues. In contrast, 41 and 32 masses were unique to the distal and proximal sections of *C. flavidus*, while 33 and 13 masses were unique to the distal and proximal sections of *C. frigidus* (Figure 4A). Despite the higher overlap of highly expressing sequences between the two species at the transcriptomic level, only 22 (13%) overlapping dominant masses was seen between the two species. This low level of overlap at the proteomic level may arise from variable transcriptomic and peptide processing of conotoxins [26,27,28,29]. Interestingly, threefold higher total number of masses were detected in the native distal proteome compared to the proximal proteome in both species (Figure 4B), but the origins of these differences were not clear.

#### Venom Duct Localisation Patterns of Dominant Peptide Masses in *C. flavidus* and *C. frigidus* and Sequence Predictions for Dominant Peptide Masses 

**Figure 5:**
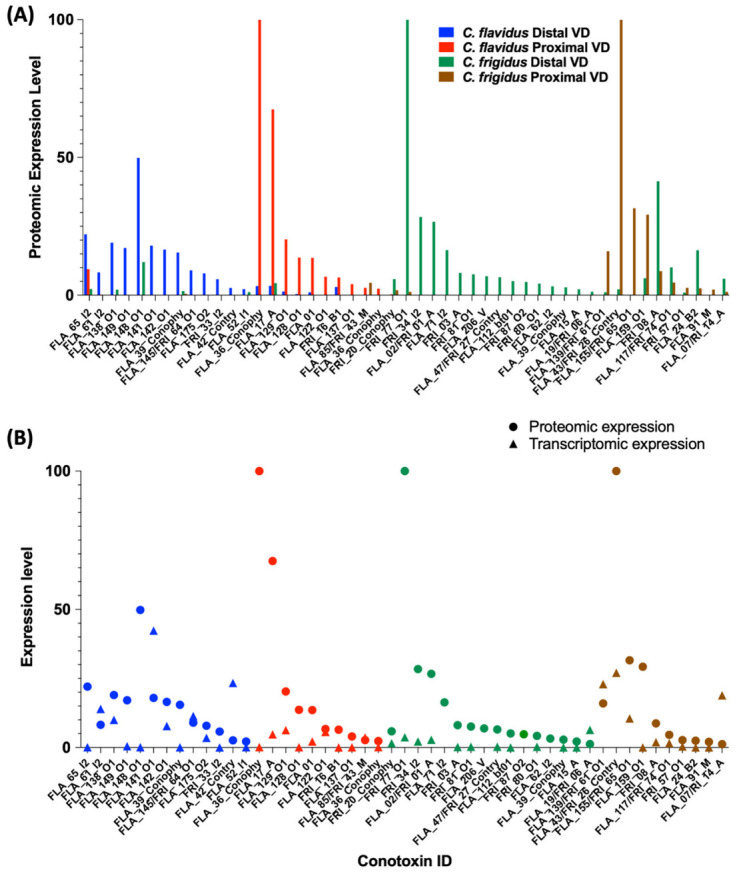
Venom duct expression of conotoxins in *C. flavidus* and *C. frigidus* predicted from transcriptomic and proteomic data. (**A**) Relative expression levels of predicted sequences in the distal and proximal venom duct proteomes of *C. flavidus* and *C. frigidus*. (**B**) Comparison of the predicted conotoxin expression levels at the transcriptomic and proteomic levels in both *C. flavidus* and *C. frigidus*.

The mass predictions of the transcriptomic sequences by introducing possible PTMs were performed, and the predicted masses were matched with the reported masses in the native extracted venoms from the duct sections. The masses with predicted sequences are shown in Table S3. Predicted sequence mass expression levels in the venom duct sections of *C. flavidus* and *C. frigidus* along with comparison to their transcriptomic expression are shown in Figure 5 (further de novo sequencing analyses were not performed to confirm the predicted sequences as a part of this study). The disparity in expression levels between the proteome and transcriptome may have arisen from intraspecific variations, as different specimens were used for transcriptomic and proteomic studies, or from distinct regulatory mechanisms associated with conotoxins translation. In the distal sections of the venom duct in both species, the number and expression level of dominant masses in the range of 3000–4000 were significantly higher compared to the respective proximal section (Figure 4B). Mass predictions for the transcriptomic sequences indicated the mass range of the O1 and I2 peptides were in the same range, and several O1 and I2 superfamily peptides were predicted for some of these dominant masses (Table S3). O1 and I2 superfamilies had the first and second highest number of sequences, respectively, in both *C. flavidus* and *C. frigidus* transcriptomes, and we can suggest that this complexity appears to have translated to the proteome. 

Interestingly, all predicted I1 and I2 peptides were localised in the distal section with little or expression in the proximal section in both species (Figure 5A, Table S3). Previous studies have shown that I1 and I2 peptides can be dominant in worm-hunting cone snails (Table S4) [18,27,30,31,32,33,34] and might be exclusively used for predation. On the other hand, predicted O1 peptides were found in both distal and proximal segments of the venom duct. However, all dominant O1 peptide sequences found in the proximal sections belonged to the O1-sub-group 1 (Figure S3), while the distal section had dominant peptides from both sub-group 1 and 2 O1 conotoxins. Interestingly, the dominant O1 toxins from sub-group 1 in the distal section were not identical to the dominant O1 conotoxins in the proximal section, suggesting these toxins may have a specific role in envenomation. Group 3 O1 conotoxins (O2) were exclusively found in *C. frigidus* transcriptome, and one (FRI_58) was also found in the distal venom duct section of *C. frigidus*. Mass predicted as FRI_77 from the O1 sub-group 2 was the most dominant in the distal proteome of *C. frigidus*. 

The most dominant mass in the *C. frigidus* proximal proteome was predicted to be a contryphan (FRI_26). A few other dominant masses were predicted to be contryphans, and FRI26 was found in both distal and proximal venom ducts of *C. flavidus* and *C. frigidus*. However, no clear localisation of the contryphans was seen in *C. flavidus* (Figure 5A). In *C. flavidus*, the highest expressing peptide masses in both distal (8378.9_60.44) and proximal (8378.9_60.44) venom duct sections were high molecular weight peptides. We could not confidently predict a sequence for 8378.9_60.44 in the distal section, while 8378.9_60.44 was predicted to be a conophysin, FLA_36_Conophy. Seven A superfamily peptides (four 4/7 and three 4/6) were found across both distal proximal venom duct sections, again with no clear localisation pattern along the duct, except that A superfamily peptide expression was higher in the distal venom duct of *C. frigidus* (both 4/7 and 4/6 types). 

### 2.4. Comparison of C. frigidus and C. flavidus Toxin Expression Patterns to Two Morphologically Similar Unidentified Sub-Groups 

During collection of *C. flavidus* and *C. frigidus* specimens from One Tree Island Reef, two cryptic sub-groups were also identified (sub-group 1 and sub-group 2 in this manuscript). On the basis of shell pattern, shell size, shell colour, and syphon and foot patterns, both sub-groups closely resembled *C. flavidus*, except sub-group 1 had a round shaped crown and sub-group 2 had a flat crown, while *C. flavidus* crown shape had an intermediate shape (Table 2). These sub-groups did not match the morphological descriptions (lip colour/pattern, the shell colour/pattern, shell shape, syphon colour) of other phylogenetically and morphologically related Virgiconus species commonly found in the Indo-Pacific region, including C. virgo, C. ermineus, and C. coelinae [35]. While the morphological features of sub-group 2 resembled the newly described C. paesei from Hawaii [16,35], more detailed morphological and molecular studies are required before the sub-groups from One Tree Island Reef can be more completely identified.

**Figure 6:**
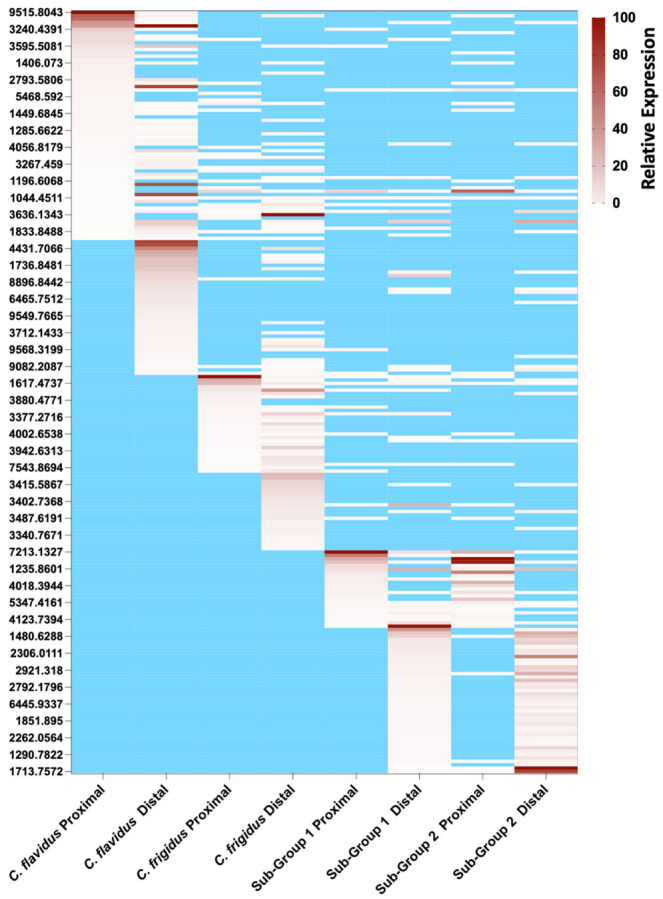
Heat map of peptide mass distribution across the proximal and distal venom ducts of *C. flavidus, C. frigidus* and two related sub-groups. Relative intensity of peptide peaks were calculated relative to the dominant peptide mass of each chromatogram (20 µg crude venom analysed). Major peptide masses (>1% relative intensity) searched across all four mass lists for mass and retention time equivalence are shown. Masses are ranked from the highest to lowest abundant starting from *C. flavidus* proximal venom through to unidentified group 2 distal venom. The mass lists, relative abundance, and predicted sequence are shown in Table S3.

To establish the similarities and difference in venom composition to *C. flavidus* and *C. frigidus*, we compared their native venom expression profiles. Dominant mass expression patterns across the distal and proximal venom duct sections (Figure 6) revealed sub-group 1 and 2 venoms were related but had little overlap to the venoms of *C. flavidus* and *C. frigidus*. Indeed, 24 and the 49 dominant masses were shared between the sub-groups in the proximal and distal duct sections. The relative expression of the dominant masses with their retention times are shown in Table S3. Surprisingly given their close morphological resemblance to *C. flavidus*, only one dominant mass in the proximal section was also found in *C. flavidus* venom (also found in *C. frigidus*), while eight minor masses were common between the two new sub-groups and *C. flavidus*, and trace levels of some dominant *C. flavidus* and *C. frigidus* masses (pale white in the heat map) were also detected in the distal and proximal proteomes of the two sub-groups. Despite these conotoxin-specific differences, at a more general level, the mass profiles of the two unidentified sub-groups showed a similar pattern to that of *C. flavidus* and *C. frigidus*, with the largest number of peptides masses found at 3000–4000 Da (Figure S11). 

## 3. Discussion

Origins of biodiversity within the *Conus* genus are associated with ecological diversification and associated adaptive radiation [2]. It is hypothesised that cone snails first evolved as worm hunters and later repurposed their defensive venoms for fish and mollusc hunting [6,36,37]. These adaptations allowed exploitation of new ecological niches [6,33,38,39], giving rise to over 800 species classified into 80 clades over ≈30 my (sub-genus) [35]. This rapid expansion of species continues, giving rise to cryptic species with little or no obvious morphological differentiation [5,15,16,19]. In this study, venom diversification between closely related *C. flavidus and C. frigidus* was investigated, revealing similar conotoxin superfamily profiles. Interestingly, *C. flavidus* venom was characterised by higher transcriptomic expression and complexity, the presence of additional minor superfamilies, and ≈2 fold more unique conotoxins. Given the collection site, tissue dissection and RNA collection and analysis were matched to reduce variability, we suggest that the differences observed likely reflect evolutionary differences between *C. flavidus* an *C. frigidus*. 

Lawler and Duda [16] predicted that *C. flavidus* and *C. frigidus* arose from a common ancestor. In support, both species were found to express the same superfamilies and shared 64% of conotoxin sequences (≈90% of the total conotoxin expression). As both species exploit similar niches and prey species (mainly sedentary *Terebellidae* polychaetes), it is reasonable they would have similar venom profiles. A comparative study between the worm hunting *C. lenavati* and *C. tribblei* of the *Splinoconus* clade also showed similar venom profiles, with the shared toxin sequences accounting for ≈50% of conotoxins in *C. tribblei* and 81% in *C. lenavati* [18]. Interestingly, the expression of species specific conotoxins was higher in *C. flavidus* and *C. tribblei* compared to *C. frigidus* and *C. lenavati,* suggesting that evolutionary pressures favoured less sequence retention and/or diversification in these latter species, perhaps due to different species-specific selection pressures. In contrast, three closely related fish hunting species (*C. catus, C. striolatus*, and *C. striatus*) of the *Pionoconus* clade showed <2% sequence identity despite having a similar superfamily profile [21,40,41]. On the basis of these findings, we suggest that diversifying selection pressures might be higher for more recently evolved fish-hunting (and likely mollusc-hunting) species compared to worm-hunting species of cone snails, reflecting adaptive divergence around new targets for capture prey and defence. Interspecific competition for limited resources may also influence expression patterns and conotoxin diversity [14,42]. Further studies on adaptive divergence rate in superfamilies and exogenome evolution across different clades of cone snails are expected to help clarify the origins of these differences. 

O1, M, and T conotoxin are widespread among cones and likely provide a minimal set of conotoxins required for the effective function of the venom [7,9,38]. Table S4 shows the venom profiles of 21 published worm-hunting cone snail transcriptomes from 12 clades widely distributed in the Indo-Pacific [18,19,27,30,31,32,33,34]. As noted in Table S4, approaches used to obtain these data differ and thus any comparisons are qualitative in nature. Comparing the relative sequence numbers found for each superfamily revealed that O1, M, T, I2, and O2 superfamilies are indeed dominant across worm hunting species. Variations in expression levels were observed in *C. tribbeli* and *C. lenavati (Kioconus)* and *C. rattus* (*Rhizoconus*), where Con-ikot-ikots dominated (Table S4), and *C. vexillum and C. miles* (*Rhizoconus*), where D superfamily conotoxins were prominent. Interestingly, these core superfamilies (O1, O2, M, and T) were also found across 14 species of endemic West African species from *Africonus*, *Varioconus*, and *Kalloconus* clades [39]. Collectively, these comparisons suggest that living cones have mostly evolved from ancestral O1, O2, M, and T superfamilies for predation and/or defence.

In *C. flavidus* and *C. frigidus* transcriptomes, the O1 superfamily was most abundant. However, these O1 superfamily conotoxins were unrelated to known ω-and δ-conotoxins from fish- (ω-MVIIA, ω-CVID, ω-GVIA, ω-TVIA, δ-PVIA, and δ-MVIA), mollusc- (ω-TxVII, ω-PnVIA, and δ-TxVIA) and worm (δ-TsVIA and δ-SuVIA)-hunting species, suggesting they evolved specifically for predation on worms. Interestingly, the O1 superfamily conotoxins from *C. flavidus* and *C. frigidus* were also unrelated to the two mammalian active ω-conotoxins MoVIA and MoVIB from vermivorous *C. moncuri* [43]. 

The I superfamily is relatively common across worm-hunting cone snail species (Table S4) [18,27,30,31,32,33,34], but less abundant in fish hunting cone snails [20,21,22,41,44], suggesting these conotoxins may have evolved for predation on worms. I superfamily sequences showed a high level of high diversity between *C. flavidus* and *C. frigidus* transcriptomes, suggesting species-specific specialisation. In the species investigated, the predatory venom of cone snails originates in the distal venom duct, while the defensive venom is expressed in the proximal venom duct [36]. Given the I superfamily peptides were mostly localised in the distal venom duct (Figure 5A), we propose they have evolved for predation on worms. A superfamily peptides are also highly expressed in the venom ducts of *C. flavidus* and *C. frigidus*, with selective expression distally or proximally depending on the specific peptide. We previously identified two A superfamily peptides (Pl169 and Pl170) with 4/7 cysteine structure from worm-hunting *C. planorbis* defensive venom [30]. Surprisingly, the *C. flavidus* and *C. frigidus* conotoxins identified showed no sequence similarity to known α-conotoxins (Figure S4). Further functional studies are needed to identify the biochemical targets of *C. flavidus* and *C. frigidus* I and A superfamily conotoxins, which are anticipated to be worm specific.

Contryphans are another major class of conotoxins widely distributed across fish- [21,36,40], mollusc- [45,46], and worm-hunting [46,47] lineages. Although the biological significance and pharmacological properties of contryphans remain to be defined, their abundance at both the transcriptomic and proteomic levels suggests they play a major role *C. flavidus* and *C. frigidus*. Previous studies on the contryphans Lo959 from vermivorous *C. loroissi* and Am975 from molluscivorous *C. amadis* suggest they target high-voltage gated calcium channels, albeit with modest potency at mammalian subtypes [46]. The contryphans found in *C. flavidus* and *C. frigidus* transcriptomes have high sequence similarity to contryphans from *C. amadis*, *C. loroisii*, *C. geographus*, *C. textile*, and *C. striatus* (Figure S6), indicating that contryphan sequences are highly conserved across all clades of cone snails, irrespective of the dietary specialisation. Interestingly, FLA_43/FRI_26 is identical to Lo959 from *C. loroissi* and abundantly expressed in the proximal venom duct of *C. frigidus* (Figure 5A), suggesting contryphans may play a role in defence, at least in worm-hunting species.

Finally, we identified two cryptic specimens closely resembling *C. flavidus* in size and shape that were collected at the same time from the second and third lagoon of the One Tree Island Reef, where *C. flavidus* and *C. frigidus* were also found (Figure S12). Despite their resemblance to *C. flavidus* morphology, the dominant mass profile of these sub-groups was most similar to each other than to *C. flavidus*, suggesting they might be the same or recently diverged species. Thus, proteomic comparisons can provided important comparative data useful in defining cone snail species. Given their related shell morphology, we predict these unidentified subgroups are also members of the *Virgiconus* clade. It is necessary to identify these species using molecular studies to further discuss and understand this significant difference in venom composition. Although gene expression levels are generally more similar among closely related taxa than among more distantly related ones [18,39], exceptions to this pattern have been reported [19,48]. When the expression patterns of four-loop conotoxin loci was compared across six related species from the *Virroconus* clade, these patterns did not show a linear correlation to phylogenetic affinity. That is, a similar four-loop conotoxin loci expression pattern was observed between *C. abbreviates* and *C. milliaris*, while recently separated *C. aristophanes* from *C. abbreviates* showed a different expression pattern, perhaps due to organismal adaptations [48]. Similarly, the recently separated cryptic species complex, *C. andremenezi* and *C. praecellens*, express an average of 64% sequences with >95% identity and only 5.5% sequences with 100% identity [19], suggesting a close relationship but also a notable species-specific divergence between these two species.

## 4. Materials and Methods

### 4.1. RNA Extraction, cDNA Library, 454 Sequencing, and Assembly

Two adult specimens of *C. flavidus* and *C. frigidus* collected from One Tree Island Reef, Queensland, were used for the transcriptomic study. Snails were sacrificed on ice, and stripped venom duct cells were placed in a 1.5 mL tube containing 1 mL of TRIzol reagent (Invitrogen, Waltham, MA, USA) prior to RNA extraction according to the manufacturer’s instructions, with mRNA being further purified using an Oligotex mRNA mini kit (Qiagen, Valencia, CA, USA). Extracted mRNA was submitted to the Australian Genomic Research Facility (AGRF), where cDNA library construction and sequencing were carried out using a Roche GS FLX Titanium sequencer from one-eighth of a plate. Finally, the sequence assembly was performed using Newbler 2.3 (Life Science, Frederick, CO, USA).

### 4.2. Conopeptide Sequence Analysis

Raw cDNA reads and Newbler-2.3-assembled isotigs were searched using the default parameters in the freely available inhouse software ConoSorter (University of Queensland, Brisbane, Australia) [49] and further analysed using SignalP4.1 (DTU Health Tech, Lyngby, Denmark) and ConoPrec (ConoServer, Brisbane, QLD, Australia) [50] to identify the conserved signal sequences, cysteine frameworks, and cleavage sites [51]. During this process, precursors less than 50 amino acids, transcripts with signal sequence hydrophobicity less than 50%, repeated sequences, truncated and elongated versions of highly expressing peptides with an odd number of cysteines, and sequences with read number less than 2 were manually removed. Considering the published variations in signal conservation within superfamilies, the cut-off value used to assign a gene superfamily was set as 53.3% [49,50].

### 4.3. Specimen Collection for Proteomics

*C. flavidus* and *C. frigidus* (20 specimens each) were collected from the second and third lagoons of the One Tree Island Reef, Queensland (Figure S12). Species identification was performed according to the identification key explained previously [35]. Two cryptic sub-groups closely resembling *C. flavidus* were also identified and separated from *C. flavidus* on the basis of the crown shape and named sub-group 1 and 2 prior to formal identification. 

### 4.4. Cone Snail Venom Peptide Extraction

Four specimens of *C. flavidus*, *C. frigidus*, and two sub-groups were sacrificed, and the venom duct was dissected. The dissected venom ducts were segmented into distal (D) and proximal (P) sections, with the extracted venom from each section using a solution of 30% acetonitrile acidified with 0.1% formic acid as previously described [26,36]. The extract was lyophilized and stored at −20 °C until use.

### 4.5. Reduction, Alkylation, and Trypsin Digestion of Extracted Venoms

A total of 100 μg of lyophilised venom was reconstituted into 50 μL of freshly prepared 100 mM NH_4_HCO_3_ in 30% acetonitrile at pH 8 prior to reduction and alkylation using the triethylphosphine/iodoethanol protocol [52]. Sigma proteomic sequencing-grade trypsin was used for enzyme digestion of reduced and alkylated peptides, as described previously [26,40]. 

### 4.6. LC-ESI-MS Analysis

Dissected venom was centrifuged (12,000× *g*) to remove particulate matter prior to liquid chromatography–electrospray mass spectrometry (LC-ESI-MS) on a Sciex TripleTOF 5600 instrument coupled to a Shimadzu 30 series HPLC system. Full scan mass spectrometric analysis and product ion MS/MS analysis using Information Dependent Acquisition (IDA) were performed on the reduced and reduced/alkylated injected venom samples. LC separation was achieved using a Zorbax C_18_ 4.6 × 150 mm column eluted with a linear 1.3% B (acetonitrile/0.1% formic acid (aq) min^−1^ gradient at a flow rate of 0.2 mL min^−1^ over 90 min. A cycle of one MS scan over *m*/*z* 300–2000 was followed by multiple tandem mass spectra (MS/MS) using a rolling collision energy relative to *m*/*z* up to a maximum of 80 eV. 

### 4.7. Proteomic Data Analysis 

For proteomic data analysis, LC-ESI-MS reconstruction was carried out using Analyst LCMS reconstruct BioTools (Framingham, MA, USA). The analysed mass range was set to 800–10,000 Da, with masses >10,000 Da excluded from further analysis. Mass tolerance was set to 0.2 Da, and the S/N threshold was set to 10. Reconstructed mass lists from LC-ESI-MS analysis of native injected and dissected venom samples were processed to remove Na^+^ and K^+^ adducts and duplicate masses using the embedded tools in ConoServer [50]. The processed LC/MS mass lists containing the monoisotopic mass, retention time, and relative intensity were used to generate the mass list for each sample. Relative mass intensities were generated as a percentage of the most abundant peptide in each individual venom sample using Analyst^TM^ (version 1.6) software (SCIEX, Framingham, MA, USA).

Masses were predicted for the resulting transcriptomic sequences using ConoMass tool 1 [50]. Then, the reconstructed mass lists were matched with predicted sequence masses using ConoMass tool 2 [50]. The precision level was set to 0.1 Da for sequence and mass search, while manual search accuracy was set to 100 ppm. Sequence matches were further explored using the ProteinPilot tools and manual inspection of the peptide expression levels in the MS and MS/MS chromatograms. 

The ProteinPilot™ 4.0 software (SCIEX, Framingham, MA, USA) was used to search the LC-ESI-MS/MS mass lists obtained at a mass tolerance of 0.05 Da for precursor ions using the reduced and reduced/alkylated samples. These masses (0.1 Da tolerance) were matched against a protein database comprising all conopeptide sequences obtained from our transcriptomic analysis. Post-translational modifications (PTM) used in the search included amidation, deamidation, hydroxylation of proline and valine, oxidation of methionine, carboxylation of glutamic acid, cyclisation of N-terminal glutamine (pyroglutamate), bromination of tryptophan, and sulfation of tyrosine and O-glycosylation. The threshold confidence level for accepting identified spectra was set to 99.

## 5. Conclusions

Sympatric *C. flavidus* and *C. frigidus* are phylogenetically and morphologically related species that likely form part of a broader species complex. To understand venom variability within this species complex, we undertook proteomic and transcriptomic studies. Transcriptomics revealed a surprising level of similarity in *C. flavidus* and *C. frigidus* venoms, with 68 common sequences accounting for 45% and 90% conotoxin expression in of *C. flavidus* and *C. frigidus*, respectively. O1 superfamily peptides dominated both the transcriptome and proteome with no clear localisation in the venom duct expression. In contrast, I2 superfamily peptides that typically dominate worm hunting cone snail venoms localised in the distal venom duct of both species, suggesting predatory roles. Interestingly, the distal venom duct had ≈3-fold higher peptide expression than the proximal duct, despite expressing similar superfamily profiles. These differences are suggestive of regulatory differences between the distal and proximal regions that are not yet understood. While *C. flavidus* and *C. frigidus* share a similar venom profile at both superfamily and conotoxin levels, diversification rates appeared higher for *C. flavidus* than *C. frigidus*. Overall, our results show that the sequence profile did not significantly change during this speciation event of *C. flavidus* and *C. frigidus*. A proteomic comparison of two possibly related sub-groups also collected from One Tree Island revealed a distinct peptide mass expression in the venom duct with a little overlap to *C. flavidus* venom, despite their morphological resemblance, suggesting a likely divergence from a common ancestor and subsequent conotoxin diversification.

## Figures and Tables

**Table 1:** *C. flavidus* and *C. frigidus* transcript analysis.

	Unique *C. flavidus* Transcripts		Unique *C. frigidus* Transcripts		Common Transcripts			All Transcripts
Superfamily	Number	Read Number (Relative Expression)	Number	Read Number (Relative Expression)	Number	*C. flavidus* Read Number (Relative Expression)	*C. frigidus* Read Number (Relative Expression)	Number
O1	29	14,539 (39.4)	11	241 (4.9)	15	16,836 (39.2)	2561 (51.9)	55
O2	8	320 (0.4)	5	52 (1.1)	9	919 (1.2)	350 (7.1)	22
O3	8	959 (1.2)	1	18 (0.4)	5	1250 (1.6)	152 (3.1)	14
I2	19	1781 (2.2)	6	95 (1.9)	4	140 (0.2)	63 (1.3)	28
I1	5	291 (0.4)	1	9 (0.2)	3	258 (0.3)	84 (1.7)	9
A	11	635 (0.8)	6	56 (1.1)	8	1930 (2.4)	462 (9.4)	25
M	9	417 (0.52)	3	8 (0.2)	9	1135 (1.4)	289 (5.9)	21
Divergent M	3	291 (0.6)	0	0	0	0	0	3
Contryphans	4	2846 (3.6)	0	0	4	2038 (2.6)	310 (6.3)	9
Conophysin	5	21 (0.03)	2	21 (0.4)	2	25 (0.03)	27 (0.6)	9
T	3	149 (1.2)	0	0	2	132 (0.2)	41 (0.8)	5
V	2	42 (0.05)	0	0	4	143 (0.2)	60 (1.2)	6
B1	2	37 (0.05)	2	11 (0.2)	0	0	0	4
B2	3	57 (0.07)	0	0	0	0	0	3
Conkunitzin	3	15 (0.02)	1	3 (0.06)	1	24 (0.03)	4 (0.08)	5
NSF1	2	12 (0.02)	1	2 (0.04)	1	30 (0.04)	9 (02)	4
NSF2	2	25 (0.03)	0	0	1	22 (0.03)	4 (0.08)	3
bt01	8	1349 (1.7)	0	0	0	0	0	8
ConoInsulin	6	20 (0.03)	0	0	0	0	0	6
P	3	17 (0.02)	0	0	0	0	0	3
SF-Mi2	3	19 (0.02)	0	0	0	0	0	3
Total	138	23,844 (48.93)	39	516 (10.3)	68	24,887 (51.07)	4502 (89.7)	245

**Table 2:** Morphology of *C. flavidus* and *C. frigidus* and two related sub-groups.

Species/Group	*C. frigidus*	*C. flavidus*	Sub-Group 1	Sub-Group 2
Shell (In the native collected form)	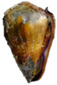	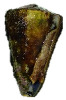	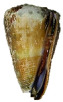	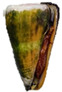
Shell length	3.5–4 cm	4.5–5 cm	4.5–5 cm	4–4.5 cm
Syphon color	Yellow and black stripes	White and black stripes	White and black stripes	White and black stripes
Crown height	45–50 mm	30–35 mm	40–45 mm	10–15 mm

## Data Availability

Raw data files of the *C. flavidus* and *C. frigidus* venom duct transcriptomes are deposited in Sequence Read Archive (SRA) of NCBI.

## Supplementary Materials

The following supporting information can be downloaded at: https://www.mdpi.com/article/10.3390/md20030209/s1, Figure S1: Sequence alignment of the New Superfamily 1 (NSF1) and 2 (NSF2) precursors. Figure S2: The superfamily expression profile of the unique (only found in one species) and common (found in both species) sequences in *C. flavidus* and *C. frigidus* transcriptomes. Figure S3: Alignment of O1 conotoxin precursors found in *C. flavidus* and *C. frigidus* transcriptome. Figure S4: I2 conotoxin precursors aligned with known I2 conotoxin precursors from other cone snails. Figure S5: A conotoxins aligned with known A conotoxins from other fish, mollusc, and worm hunters. Figure S6: Contryphan precursors aligned with known Contryphan precursors from other fish, mollusc, and worm hunters. Figure S7: TIC of the *C. flavidus* proximal venom duct showing dominant masses eluting under major peaks. Figure S8: TIC of the *C. flavidus* distal venom duct showing dominant masses eluting under major peaks. Figure S9: TIC of the *C. frigidus* proximal venom duct showing dominant masses eluting under major peaks. Figure S10: TIC of the *C. frigidus* distal venom duct showing dominant masses eluting under major peaks. Figure S11: Mass distribution ranges in the reconstructed major masses of proximal and distal venom duct extracts of Sub-groups 1 and 2. Figure S12: One Tree Island Map. Table S1: Summary of the *C. flavidus* and *C. frigidus* venom duct transcriptomes. Table S2. Integration of the transcriptome and proteomic data using ProteinPilotTM tool. Table S3: Comparative expression levels of major peptides masses detected in the MS spectrum of the separate distal and proximal venom duct sections of *C. flavidus*, *C. frigidus*, and two unidentified specimens. Table S4: Superfamily distribution in the venom duct transcriptomes of worm hunting cone snails.
