# Supplementary material for: Comparative Venomics of C. flavidus and C. frigidus and Closely Related Vermivorous Cone Snails
Source: Mar Drugs. 2022 Mar 15;20(3):209. doi: 10.3390/md20030209 (PMC8951504; doi:10.3390/md20030209)
Supplement: Supplementary file 1 [file marinedrugs-20-00209-s001.zip › Supplementary Figures.pdf]

Supplementary Figures

Comparative venomics of *C. flavidus* and *C. frigidus* and closely related vermivorous cone snails.

Himaya S.W.A.[1,*], Alexander Arkhipov[1,2], Wai Ying Yum [1] and Richard J. Lewis[1,*]

[1] Institute for Molecular Bioscience, The University of Queensland, St Lucia, QLD 4072, Brisbane, Australia
[2] Current address - School of Agriculture and Food Science, The University of Queensland, St Lucia, QLD 4072, Brisbane, Australia
* Correspondence to: R.J.L. r.lewis@uq.edu.au; Tel.: +61 7 334 62984; H.S.W.A. h.sddhihalu@imb.uq,edu.au; Tel.: +61 7 334 62722

```
                    1       10        20        30        40        50        60        70        81
                    |--------+---------+---------+---------+---------+---------+---------+---------+|
       FLA_102_NSF1   MKIGLIICLLLIAFMNGD-GSLGNTMHSRKSAGVVAAIKRYQKNFLRRSCNNCPEEPCCYGDQCMADPGHEPFCGN
       FLA_103_NSF1  MMKIGLIICLLLIAFMNGD-GSLGNTMHSRKSAGVVAAIKRFQKNFLRRTCTNCPEEPCCYGDQCMPDPGHEPFCGN
FLA_104-FRI_54_NSF1  MMKISLIICLLLIAFMNGD-GSLGNTMHSRKSAGVVAAIKRFQKNFLRRTCTNCPEEPCCYGDQCMPDPGQEPFCGN
       FLA_105_NSF2 MRVYLLLTVTLLLTSFTGADAGPMPANGHFVRRDCQWGCY-----GCHNRAGCCCGDQVCVDNNRCEPRPSQT
FLA_106-FRI_56_NSF2 MRVYLLLTVTLLLTSFTGADAGPMPANGHFVRRDCQWGCY-----GCRNRAGCCCGDQVCVDNNRCERRPSQT
       FLA_107_NSF2 MRVYLLLTVTLLLTSFTGADAGPMPANGHFVRRDCQWGCY-----GCRNRAGCCCGDQVCVDNNRCESRSSQTTTRPTQHL
          Consensus mrvyllLt!tLLLtsFtgaDaGp$pangHfvrrdcqwgc!.....gc.nRagccCg##vCvdn#rCe.rpsqt........
```

Figure S1: Sequence alignment of the New Superfamily 1 (NSF1) and 2 (NSF2) precursors

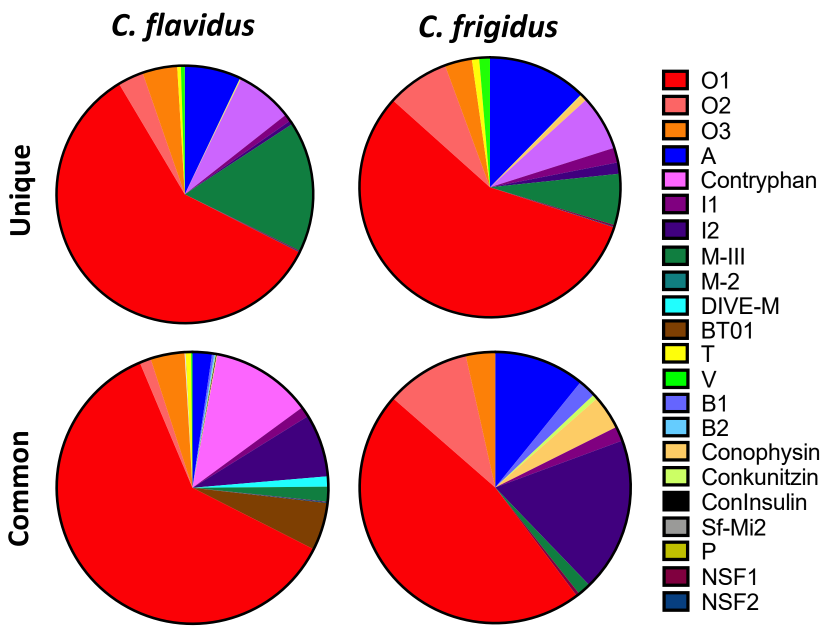

Figure S2: The superfamily expression profile of the unique (only found in one species) and common (found in both species) sequences in *C. flavidus* and *C. frigidus* transcriptomes.

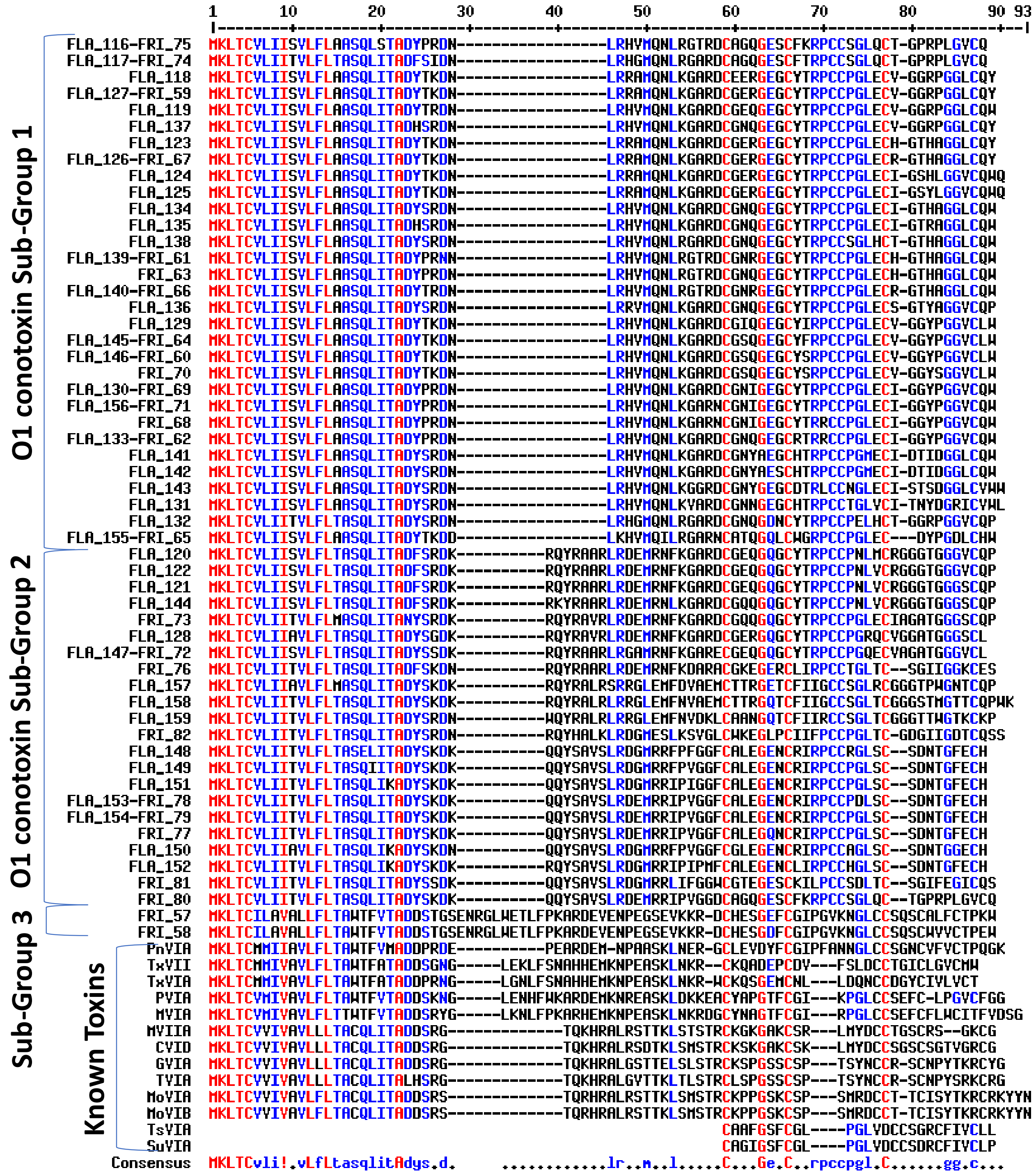

Figure S3 : Alignment of O1 conotoxin precursors found in *C. flavidus* and *C. frigidus* transcriptomes. The precursor sequences were classified into three subgroups based on the sequence similarity. Known O1 superfamily ω- and δ-conotoxins from fish, mollusk and worm hunting cone snails were aligned with the *C. flavidus* and *C. frigidus* O1 precursors using Clustal Omega. Conserved amino acids are highlighted and the consensus sequence shown at the bottom.

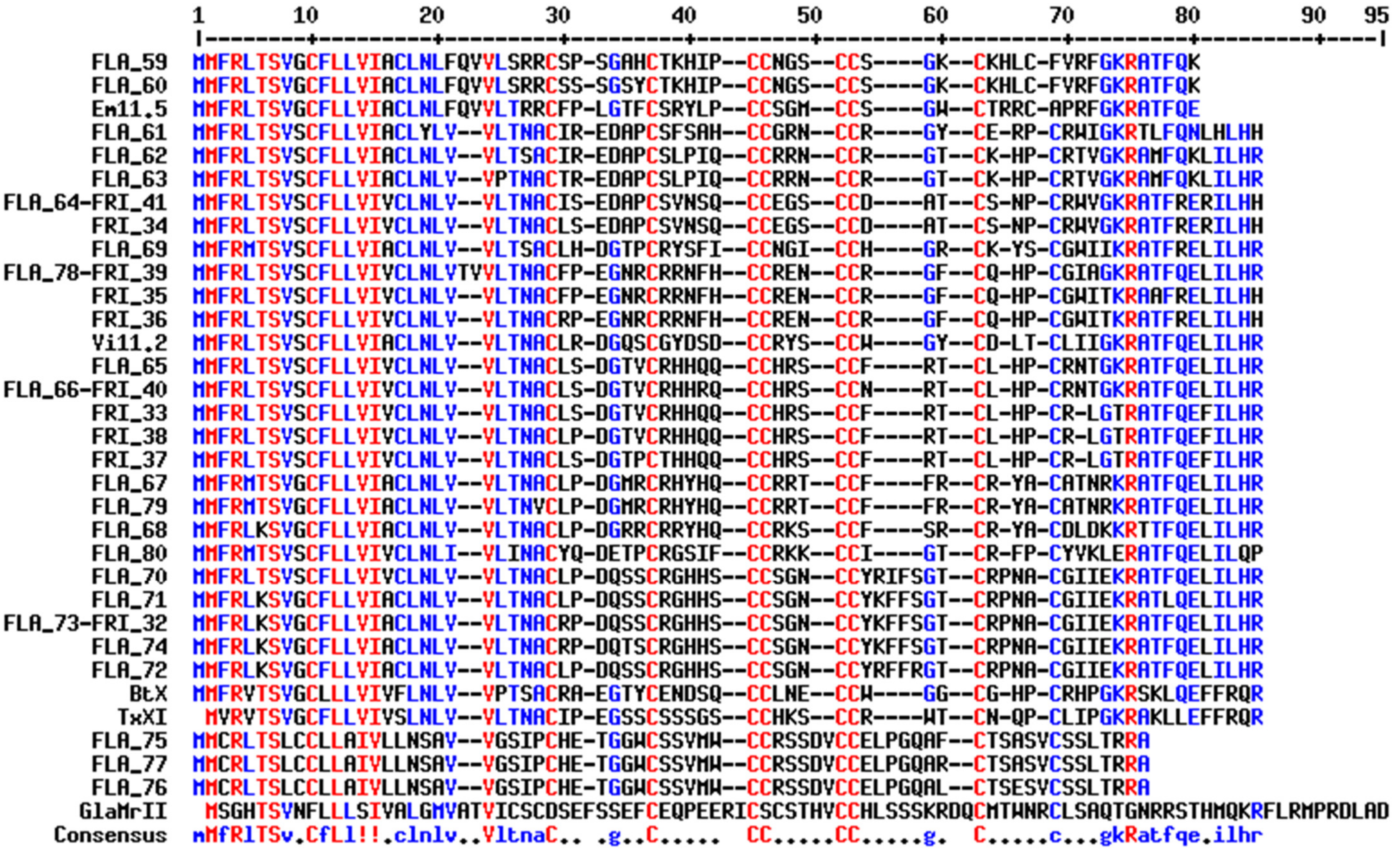

Figure S4 : I2 conotoxin precursors aligned with known I2 conotoxin precursors from other Cone snails (Vi11.2, Btx, TxXI and GlaMrII).

```
               1       10         2022
               |--------+---------+-|
       FLA_01       DCCNDPACAVNHQDLCT
FLA_02-FRI_01       DCCNDPACAVNNPGLCT
       FLA_03       DCCNDPACTVNNPGLCT
       FRI_02       DCCNLPACAASNPGLCT
       FRI_03       DCCNFPACAASNPGLCT
        Vc1.1       GCCSDPRCNYDHPEIC
         PeIA       GCCSHPACSVNHPELC
         LvIA       GCCSHPACNVDHPEIC
         LsIA      SGCCSNPACRVNNPNIC
         TxIA       GCCSRPPCIANNPDLC
          GIC       GCCSHPACAGNNQHIC
FLA_04-FRI-04       DCCSSAACAHHTPSC
       FLA_05       DCCSSVACAHHTPSC
FLA_07-FRI_14       GCCSDPPCAHHNPAC
       FRI_08       GCCSDPPCAHHNPSC
       FLA_13       GCCSNPPCAHHNPAC
       FRI_11       GCCSNPPCAHHNPDC
       FRI_13       GCCSDPPCAHHNPDC
       FLA_10       GCCSDPPCAHNNPAC
       FLA_12       GCCSDPPCARHNPAC
FLA_11-FRI_05       GCCSDPPCAHNNPDC
FLA_16-FRI_12       GCCSNPPCAHNNPDC
FLA_19-FRI_06       DCCSDPPCAHNNPDC
         ViIA      RDCCSNPPCAHNNPDCR
       FRI_10       DCCSDSPCAHNNPDC
       FLA_14       GCCSNPPCAHINPDC
       FLA_15       GCCSNPPCAHLNPAC
       FLA_17       GCCSNPPCALNNPDC
       FLA_18       GCCSSPPCARLNPAC
       FLA_06       GCCSDP-CAHKHC
FLA_08-FRI_09       GCCSDPPCAHKHC
FLA_09-FRI_07       GCCSDPPCAHKYC
         RgIA       GCCSDPRCRYRCR
          ImI       GCCSDPRCAWRC
        Pl169  TVIMHNCCTRSFCKRIYPDLCS
        Pl170   GIGGSCCVIRSCAIKFSTLCG
           SI        ICCNPACGPKYSC
           MI       GRCCHPACGKNYSC
          CIA      NGRCCHPACGKHFSC
    Consensus      .gcCs.p.Ca..np....
```

Figure S5: A conotoxins aligned with known A conotoxins from other fish (SI, MI, CIA, GIC), mollusk (TxIA) and worm hunters (PeIA, LvIA, LsIA, ViIA, RgIA, ImI, Pl169, Pl170).

```
                    1       10        20        30        40        50        60 63
                    |--------+---------+---------+---------+---------+---------+--|
           FLA_42   MGKLTILVLVAAVLLSTQVMVQGDRDQPADRDAVPRDDKPGGTSGKFMNVLRRSGCPFDPWCG
    FLA_43-FRI_26   MGKLTILVLVAAVLLSTQVMVQGDRDQPADRDAVPRDDKPGGTSGKFMNVLRRSGCPWDPWCG
   Contryphan-FR1   MGKLTILVLVAAVLLSTQVMVQGDRDQPADRDAVPRDDKPGGTSGKFMNVLRRSGCPWDPWCG
           FLA_44   MGKLTILVLVAAVLLSTQVMVQGDRDQPADRDAVPRDDKPGGTSGKFMNVLRRSGCPWDSWCG
 Contryphan-AM975   MGKLTILVLVAAALLSTQVMVQGDGDQPADRDAVPRDDNPSGMSGKFMNVLRRAGCPWDPWCG
           FLA_45   MGKLTILLLVAAVLLSTQVMVQGDRDQPTDRDAVPRDDKPGGTSGKFMNVLRRSGCTSHPWCG
           FLA_46   MGKLTILVLVAAVLLSTQAMVQGDRDQPADRDAVPRDDKPGGTSGKHMNVLRWSECAWDPWCG
    FLA_47-FRI_27   MGKLTILVLVAAVLLSTQAMVQGDRDQPADRDAVPRDDKPGGTSGKHMNVLRWSECPWDPWCG
    FLA_48-FRI_25   MGKLTILVLVAAVLLSTQAMIQGDRDQPADRDAVPRDDKPGGTSGKHMNVLRWSECPWDPWCA
    FLA_49-FRI_24   MGKLTILVLVAAVLLSTQAMIQGDRDQPADRDAVPRDDKPGGTSGKHMNVLRWSECPWDPWCV
           FLA_50   MGKLTILVLVAAVLLSTQAMIQGDRDQPADRDAVPRDDKPGGTSGKHMNVLRWSECPWDSWCV
     Contryohan-G   MGKLTILVLVAAVLLSTQAMVQGDGDQPAARNAVPRDDNPDGPSAKFMNVQRRSGCPWEPWCG
     Contryohan-P   MGKLTILVLVAAVLLSTQVMVQGDGDQPAYRNAAPRDDNPGGAIGKFMNVLRRSGCPWDPWCG
     Contryohan-S   MEKLTILVLVAAVLLSTQVMVQGDADQPADRDAVPRDDNAGGTDGKFMNVQRRSGCPWEPWCG
    Contryohan-Tx   MGKLTILVLVAVALLSTQVMVQGDGDQPADRDAVPRDDNPGGMSEKFLNALQRRGCPWQPYCG
 Leu-ContryphanTx   MGKLTILVLVAAVLLSAQVMVQGDGDQPADRKAVPREDNPGGASGKLMDVLRPKKCVLYPWCG
   Contryphan-Lo1   MGKLTILVLVAAVLLSTQAMVQ---DQPADRNAVRRNDNPGGASRKSINVLHR-GCPWDPWCG
        Consensus   MGKLTILVLVAavLLStQvMVQgd.DQPAdRdAVpR#DnPgG.sgKfm#vlrrsgCpwdPwCG
```

Figure S6: Contryphan precursors aligned with known Contryphan precursors from other fish (Contryphan-G, Contryphan-S Contryphan-P), mollusk (Contryphan-Tx, Leu-Contryphan-Tx) and worm (Contryphan-Lo1) hunters.

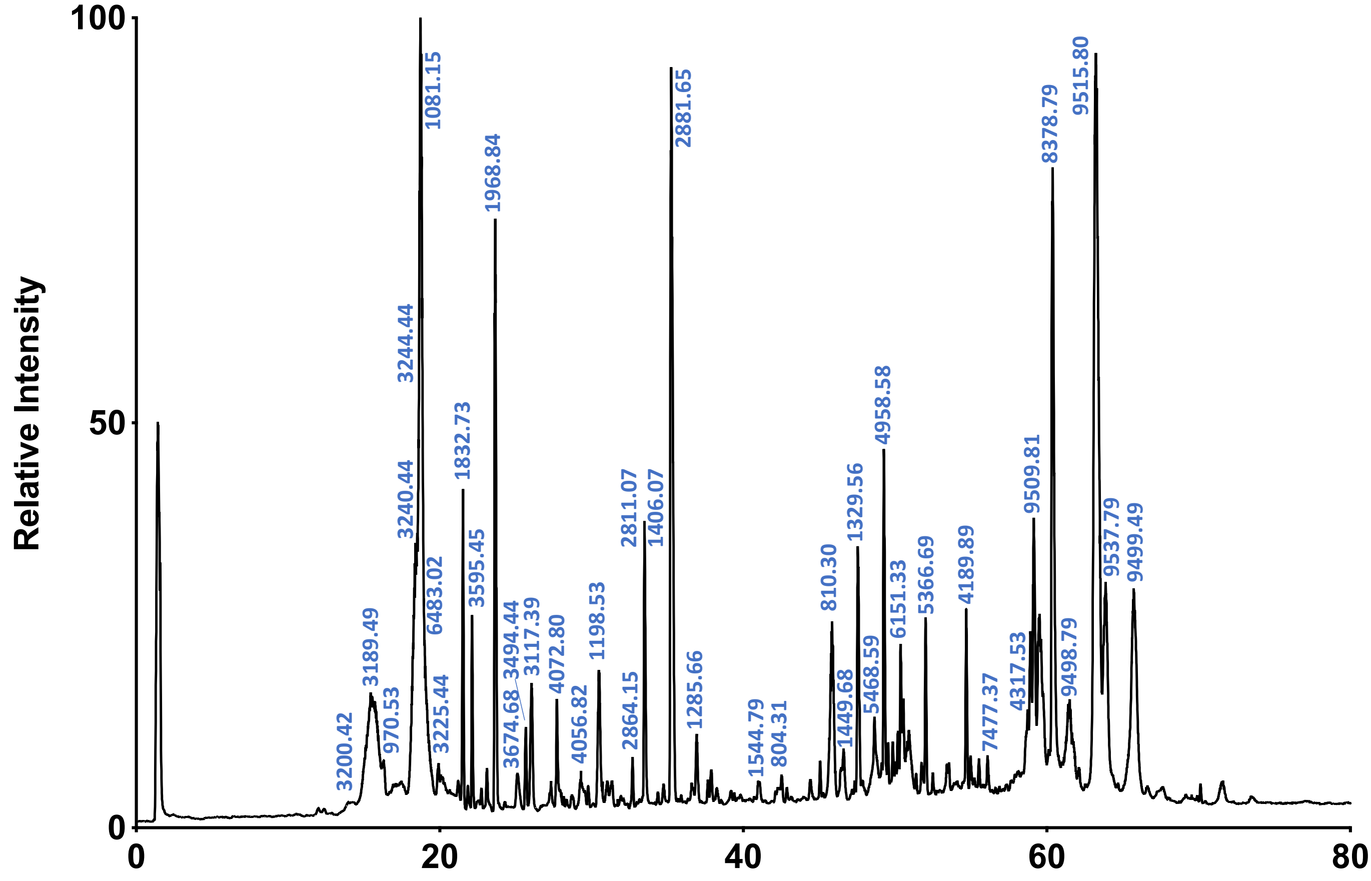

Figure S7: TIC of the *C. flavidus* Proximal venom duct showing dominant masses eluting under major peaks.

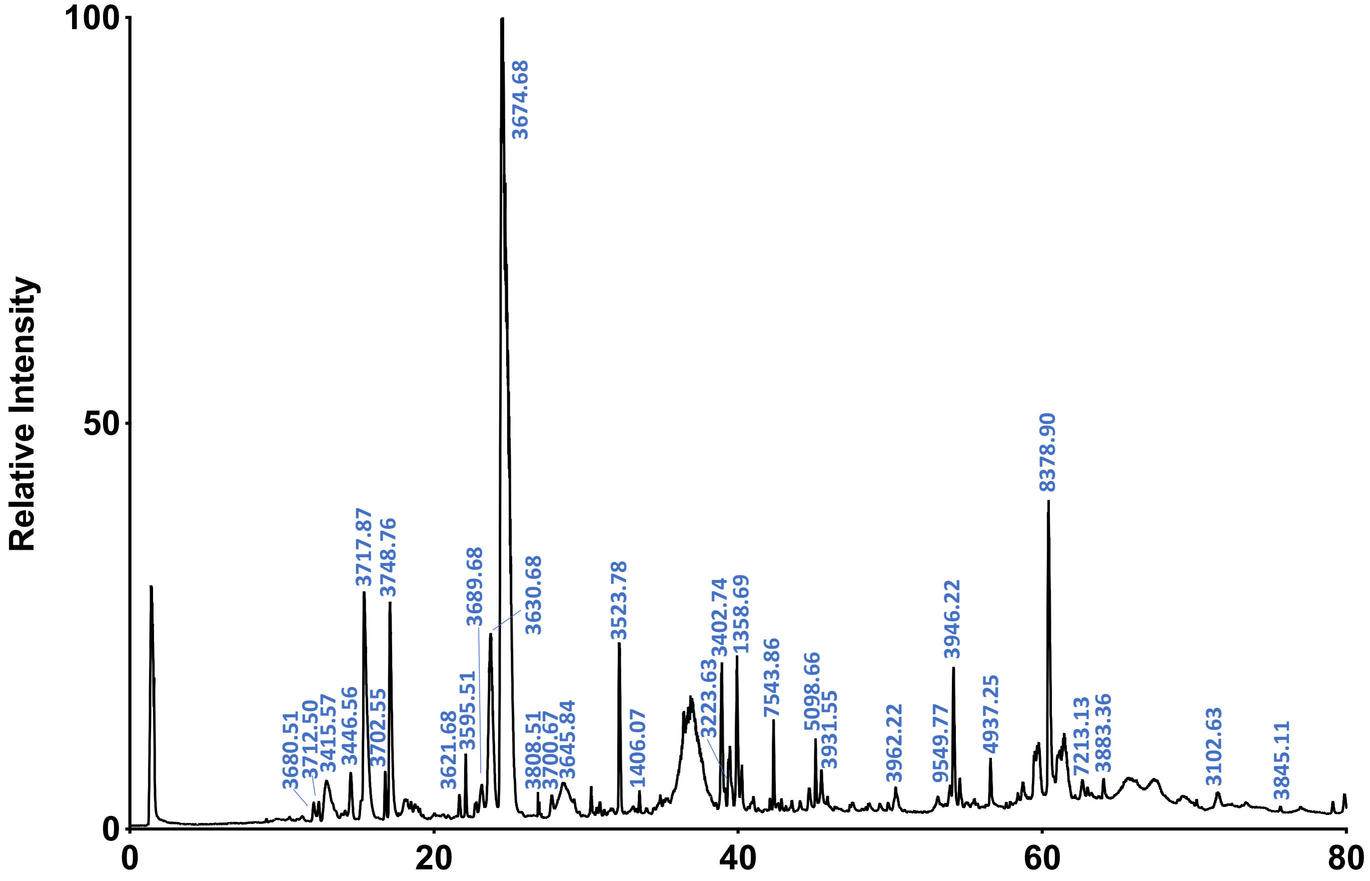

Figure S8: TIC of the *C. flavidus* Distal venom duct showing dominant masses eluting under major peaks.

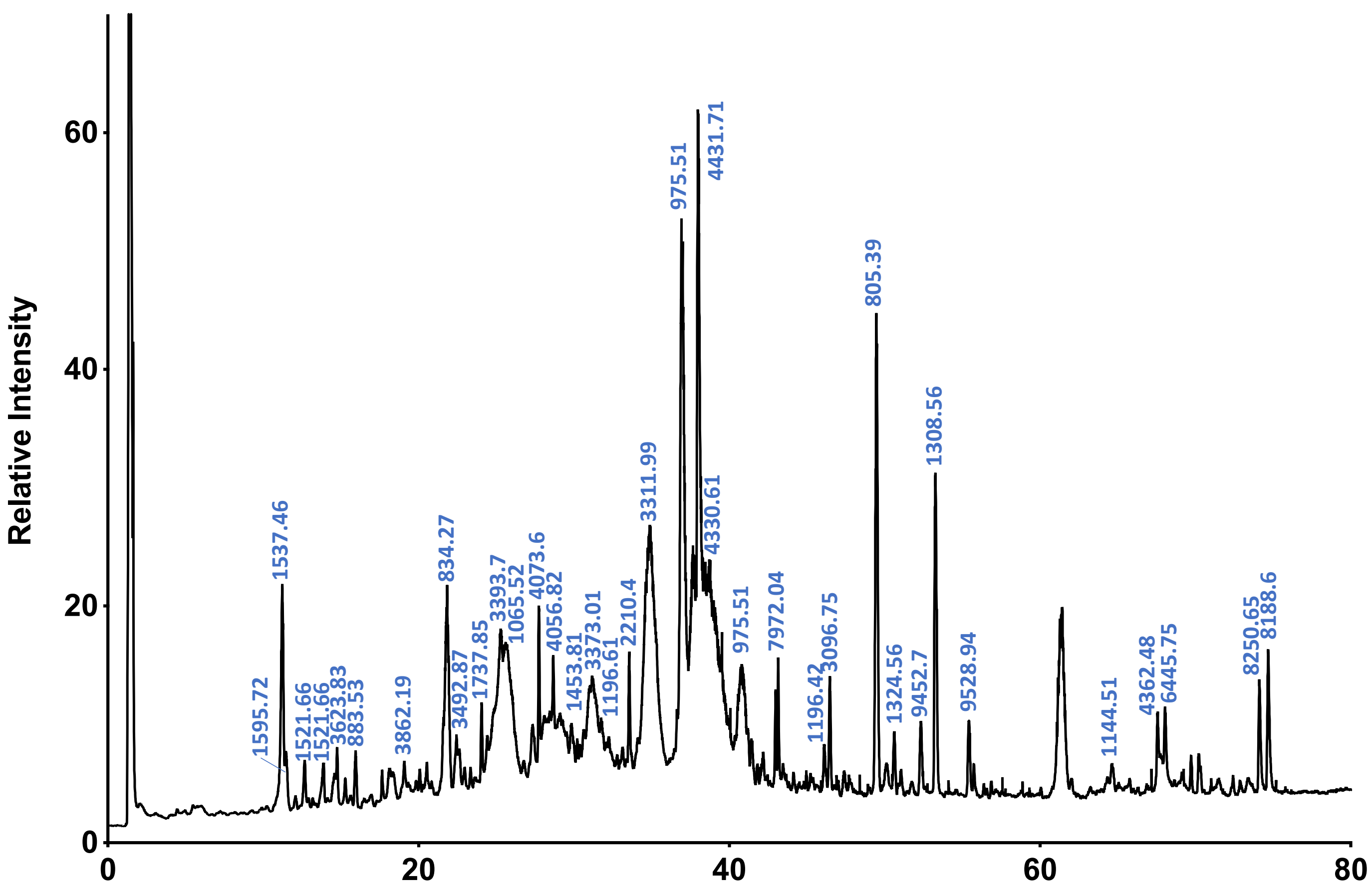

Figure S9: TIC of the *C. frigidus* Proximal venom duct showing dominant masses eluting under major peaks.

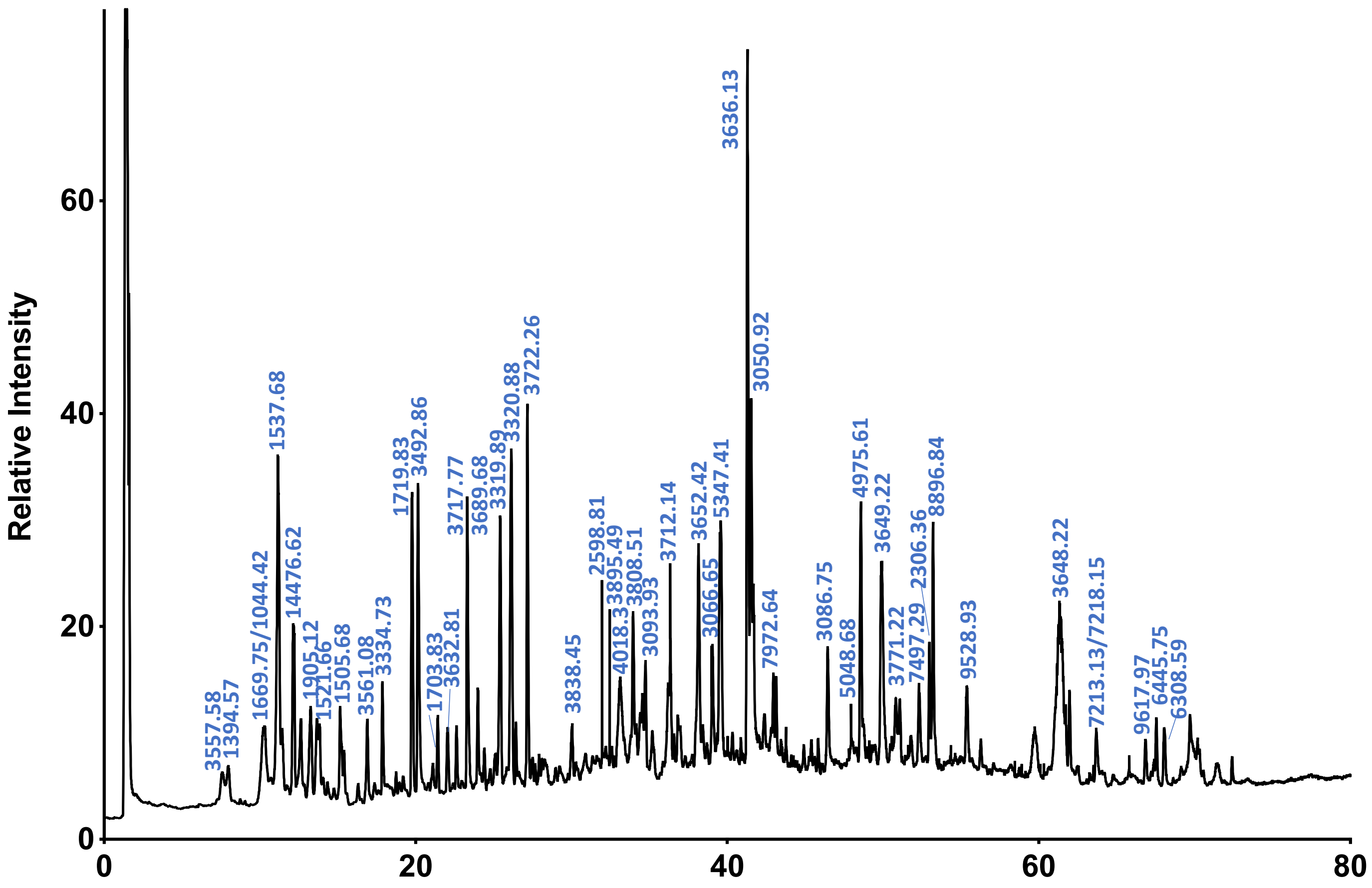

Figure S10: TIC of the *C. frigidus* Distal venom duct showing dominant masses eluting under major peaks.

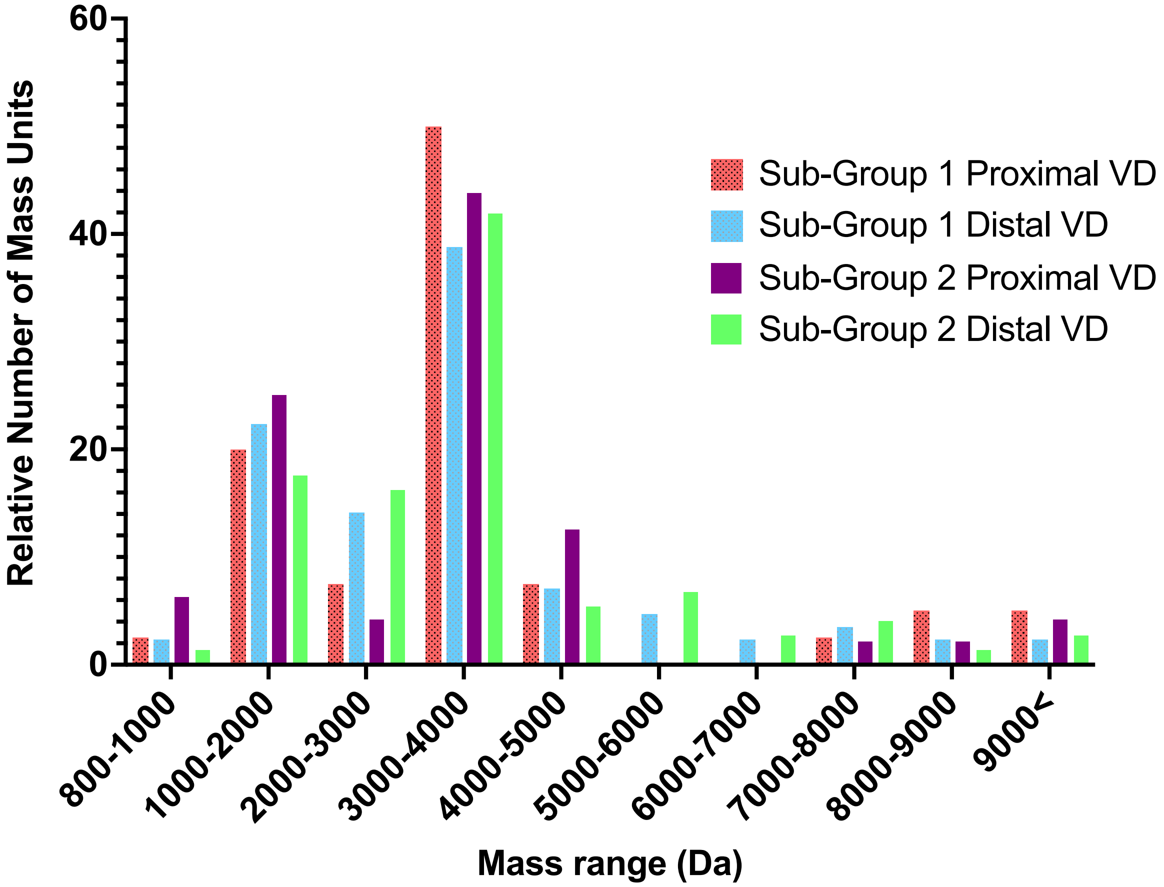

Figure S11: Mass distribution ranges in the reconstructed major masses of proximal and distal venom duct extracts of sub-groups 1 and 2.

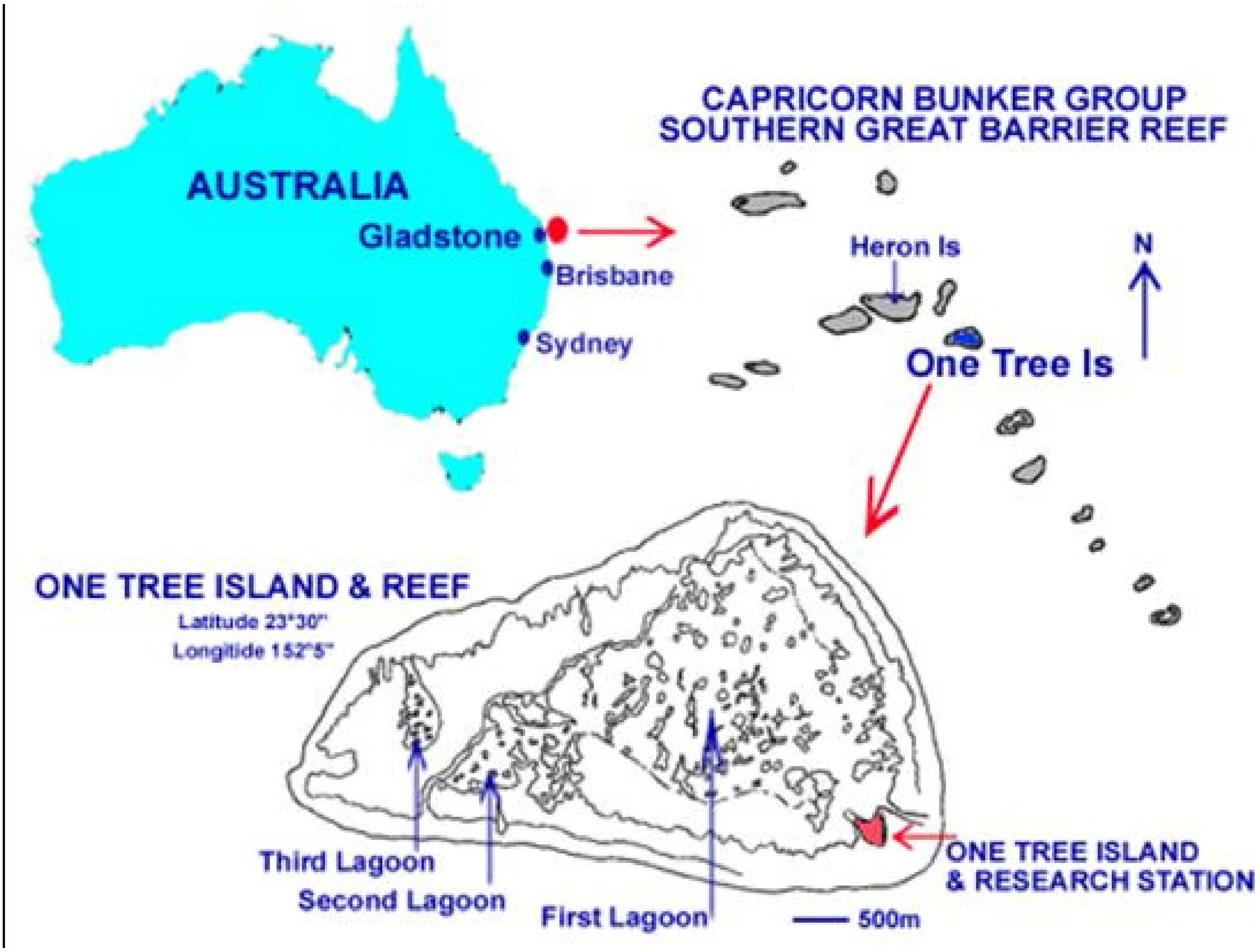

Figure S12. One Tree Island Map. The map is taken from the One Tree Island Research Station resources.
