# Supplementary material for: Comparative Venomics of C. flavidus and C. frigidus and Closely Related Vermivorous Cone Snails
Source: Mar Drugs. 2022 Mar 15;20(3):209. doi: 10.3390/md20030209 (PMC8951504; doi:10.3390/md20030209)
Supplement: Supplementary file 1 [file marinedrugs-20-00209-s001.zip › Table S2.pdf]

Table S2. Integration of the transcriptome and proteomic data using ProteinPilot^{TM} tool.
Underlined sequences are matched to 99% confidence to the MS/MS fragments obtained from reduced-alkylated and reduced-alkylated-trypsin digested extracted venom from *C. flavidus* (FLA), *C. frigidus* (FRI) collected from One Tree Island reef.

.

Sequence ID	Seq. Freq.	Mature Sequence	Cystein Mod.	FLA	FRI
FLA_01_A	155	**DCCNDPACAVNHQDLCT**	Y	✓	✓
FLA_05_A	4	**DCCSSVACAHHTPS**C	Y	✓	0
FLA_12_A	2	**GCCSDPPCARHNP**AC	Y	✓	0
FLA_14_A	2	**GCCSNPPCAHINPDC**	Y	✓	0
FLA_15_A*	8	**GCCSNPPCAHLNPAC**	Y	✓	✓
FLA_17_A	336	**GCCSNPPCALNNPDC**	Y	✓	0
FLA_22_B1	19	AADENLEERR**GHSNVDKSAFDSESDPGAFMK**F**LEDNGNK**<br>**LPFEE**MDG**ASPDLGNFEPSAENDHEENKFR**FFDQQP	N	✓	0
FLA_23_B2	12	AADENLEER**REHAKFQADKSAFDSESSPEDFMKFLEENGN**<br>**TNPFEDASPDLGKFESDQNGQEENKFSFFNQQP**	N	✓	0
FLA_24_B2	26	AADENLEER**REHAKFQADKSAFDSESSPEDFMKFLEENGN**<br>**TNPFEDASPDLGKFESDQNGQEENK**FRFFNQQP	N	✓	0
FLA_31_ConIns	3	SIGTEQTCTPDEPADPQGRCGPALSQYHERMCELEEIRHGG<br>TFDPQKKRGRASLLRKRRGFLSMLKARAKRNEAGSLQRAGR<br>G**IVCECCQNH**CSDEEITEYCPPVTESSS	Y	✓	0
FLA_33_ConIns	3	TCEPEDPPDPQGK**CGPALSQYHER**MCELEEIRHGGTFDPQ<br>KKRGRVSSLRKRRGFLSMLKARAKRNEAGSLQRAGRGIICEC<br>CQNRCSDEEFTEYCPPVTESSS	Y	✓	0
FLA_34_ConIns*	2	TCEPEDPPDPQGRCGPALSQYHERMCELEEIRHGGTFDPQ<br>KKRGRASP**LR**KRRGFLSMLKARAKRNEAGSLQRAGR**GIICE**<br>**CCQNR**CSDEEITEYCPPVTESSS	Y	✓	0
FLA_35_Conophy	2	SIEQCRYCSFGKCVWPIVCCGNSGCEIGTVEANTCRPEAGQ<br>PFPCKVIGK**SCVMNNPSDPSNYYGHCIDADGR**GICCVDDN<br>TCTAHSVCP	Y	✓	0
FLA_36_Conophy	9	STECWYCSFGKCVWPIICCGDDGCEMGTAEANSCRPEGDQ<br>HFPCR**VIGKSCIMNNPSNPSNYYGHCIGADGLGICCVDDD**<br>**TCTLHSGCP**	Y	✓	0
FLA_37_Conophy	2	STECWYCSFGKCVWPIICCGDDGCEMGTAEANTCRPEGDQ<br>HFPCR**VIGKSCIMNNPSNPSNYYGHCIGADGLGICCVDDD**<br>**TCTLHSGCP**	Y	✓	0
FLA_38_Conophy	3	STEECWSCSFGK**CVWPIVCCGDDGCEMGTAEADKCRPEG**<br>**GQSFPCR**VTGQYCFMNNPSNPRNYHGHCVGGDGTGICCV<br>DDNTCTSHSGCP	Y	✓	0
FLA_39_Conophy	5	STEQCWYCSFGKCVWPIICCGDDGCEMGTAEANSCRPEGD<br>QRFPCRVIGK**SCIMNNPSNPDDHVGNCIGGDGLGICCVDD**<br>**NTCASHSGCL**	Y	✓	0
FLA_51_I1	18	**GTCSGIGQGCIHHLNCCWDMCCYGHTCVVNIIGCPPH**	Y	✓	0
FLA_52_I1	8	**GTCSGIGQGCIHHLSCCWDMCCYGHTCVVNIIGCPPH**	Y	✓	0
FLA_53_I1*	25	TCSDIGQGCTR**HLNCCWDMCCYGHTCVMNIIGCPPH**	Y	✓	✓
FLA_54_I1	136	QFFCPDSENDPLNCIETKASDATCMKSNSDQTYSYVCGYCG<br>QKK**ESCFGNKVPVSDYNCQTRK**IVNNCGGSAL	Y	✓	0
FLA_59_I2	32	**CSPSGAHCTKHIPCCNGSCCSGKCKHLCFVRF**	Y	✓	0
FLA_60_I2	30	**CSSSGSYCTKHIPCCNGSCCSGKCKHLCFVRF**	Y	✓	0
FLA_61_I2	990	**CIREDAPCSFSAHCCGRNCCRGYCERPCR**WIGKRTLFQKSP<br>PTSLKCCTEVCCFSS	Y	✓	0
FLA_63_I2*	20	**CTREDAPCSLPIQCCR**RNCCRGTCKHPCRTV	Y	✓	✓
FLA_65_I2*	8	**CLSDGTVCRHHQQCCHR**SCCFRTCLHPCRNT	Y	✓	✓

FLA_69_I2	109	CLHDGTPCR**YSFICCNGICCHGR**CKYSCGWII	Y	✓	0
FLA_77_I2*	203	IPCHETGGWCSSVMWCCR**SSDVCCELPGQAR**CTSASVCSSLT	Y	✓	✓
FLA_79_I2	9	**TNVCLPDGMR**CRHYHQCCRRTCCFFRCRYACATN	Y	✓	0
FLA_80_I2	211	**CYQDETPCRGSIFCCRKKCCIGTCRFPCYVK**LERATFQELILQP	Y	✓	0
FLA_94_M	47	**CCSRDCSVCIPCCPYGSP**	Y	✓	0
FLA_100_M	2	**QQVCCDPDVCDGGCYTCC**	Y	✓	0
FLA_107_NSF2*	23	**DCQWGCVGCRNRAGCCCGDQVCVDNNR**CESRSSQTTTRPTQHL	x	✓	✓
FLA_112_bt01	22	EGAVNIFPCNPGRCACLPME**GTSTSYQCQSIHSSTANCVGNECITESGW**	Y	✓	0
FLA_113_bt01	13	EGAVNIFPCNPGRCACLPRE**GTSTSYQCQSIHTSTANCVGNECITEDEW**	Y	✓	0
FLA_120_O1	2	**DCGEQGQGCYTRPCCPNLMCRGGGTGGGVCQP**	Y	✓	0
FLA_122_O1	400	**DCGEQGQGCYTRPCCPNLVCRGGGTGGGVCQP**	Y	✓	0
FLA_123_O1*	56	DCGERGE**GCYTRPCCPGLECHGTHAGGLCQY**	Y	✓	✓
FLA_125_O1	8	DCGERGEGCYTRPCCPGLE**CIGSYLGGVCQWQ**	Y	✓	0
FLA_128_O1	7	**DCGERGQGCYTRPCCPGR**QCVGGATGGGSCL	Y	✓	0
FLA_131_O1	368	**DCGNNGEGCHTRPCCTGLVCITNYDGR**ICVWL	Y	✓	0
FLA_135_O1	3697	**DCGNQGEGCYTRPCCPGLECIGTR**AGGLCQW	Y	✓	0
FLA_136_O1	989	**DCGNQGEGCYTRPCCPGLECSGTYAGGVCQP**	Y	✓	0
FLA_137_O1	5	**DCGNQGEGCYTRPCCPGLECVGGRPGGLCQY**	Y	✓	0
FLA_138_O1	707	**DCGNQGEGCYTRPCCSGLHCTGTHAGGLCQW**	Y	✓	0
FLA_144_O1	87	**DCGQQGQGCYTRPCCPNLVCRGGGTGGGSCQP**	Y	✓	0
FLA_148_O1	7	**FPFGGFCALEGENCR**IRPCCRGLSCSDNTGFECH	Y	✓	0
FLA_149_O1	32	**FPVGGFCALEGENCRIRPCCPGLSCSDNTGFECH**	Y	✓	0
FLA_150_O1	25	**FPVGGFCGLEGENCRIRPCCAGLSCSDNTGGECH**	Y	✓	0
FLA_151_O1	26	**IPIGGFCALEGENCRIRPCCPGLSCSDNTGFECH**	Y	✓	0
FLA_152_O1	26	**IPIPMFCALEGENCLIRPCCHGLSCSDNTGFECH**	Y	✓	0
FLA_157_O1	118	R**GLEMFDVAEMCTTRGETCFIIGCCSGLR**CGGGTPWGNTCQP	Y	✓	0
FLA_159_O1	30	R**GLEMFNVDKLCAANGQTCFIIRCCSGLTCGGGTTWGTKCKP**	Y	✓	0
FLA_177_O3	405	AVDEECKEYCEQQNKNCCGK**TNGEPVCAQTCL**	Y	✓	0
FLA_180_O3	58	**TADEACKEYCEERNKNCCGR**TNGEPVCAQLCF	Y	✓	0
FLA_187_O3	19	TTEECKEYCEQQNK**NCCGLTNGEPR**CASMCV	Y	✓	0
FLA_188_O3	56	TTEECNEYCEQQNK**NCCGLTNGEPR**CASMCL	Y	✓	0
FLA_190_P*	2	**YSCARPCDYHNDCPTSCPYCTPFQRCM**	Y	✓	✓
FLA_191_P*	9	**YSCARPCGYHNDCPTSCPYCTPFQRCM**	Y	✓	✓
FLA_198_T*	8	**HCCPIDLPCCPI**	Y	✓	✓
FLA_199_T	139	**HCCPIDLPCCPL**	Y	✓	0
FLA_203_V	4	R**DCTTCGGEECCGR**CTCTWGDNCDCVAW	Y	✓	0
FLA_02_A / FRI_01_A^*	43/31	**DCCNDPACAVNNPGLCT**	Y	✓	✓
FLA_07_A / FRI_14_A*	575/208	**GCCSDPPCAHHNPAC**	Y	0	✓
FLA_08_A / FRI_09_A^*	462/4	**GCCSDPPCAHKHC**	Y	✓	✓
FLA_09_A / FRI_07_A*	104/66	**GCCSDPPCAHKYC**	Y	0	✓
FLA_11_A / FRI_05_A^*	402/72	**GCCSDPPCAHNNPDC**	Y	✓	✓
FLA_16_A/ FRI_12_A*	240/11	**GCCSNPPCAHNNPDC**	Y	0	✓
FLA_19_A / FRI_06_A^	104/70	**DCCSDPPCAHNNPDC**	Y	✓	0
FLA_40_Conophy / FRI_21_Conophy*	4/15	STEQCWYCSFGKCVWPIVCCGSGGCQIGTAEANTCRPPAGERFPCKVIGK**SCVMNNPSDPSNYYGHCIGAEGHGVCCVDDNTCTAHSVCP**	Y	0	✓

FLA_41_Conophy / FRI_22_Conophy*	11/21	STEQCWYCSFGKCVWPIVCCGSGGCQIGTAEANTCRPQAGERFPCKVMGK**SCVMNNPSDPSNYYGHCIGAEGYGVCCVDDNTCTAHSVCP**	Y	0	✓
FLA_55_I1 / FRI_28_I1*	148/70	**QFFCPDSENDPLNCVETKPTAATCMHSRS**DK**TYSYVCGYCGQK**KES**CFGNKVPVSDYSCQSRKIVNNCGGSAL**	Y	0	✓
FLA_57_I1 / FRI_31_I1*	66/4	**QFFCPDSENDPLNCVETKPTSATCMHSRSDKTYSYVCGYCGQKKESCFGNKVPVSDYSCQSRKIVNNCGGSAL**	Y	0	✓
FLA_64_I2 / FRI_41_I2*	11/21	CISEDAPCSVNSQCCE**GSCCDATCSNPCRWV**	Y	0	✓
FLA_66_I2 / FRI_40_I2^	100/3	**CLSDGTVCR**HHRQCCHRSCCNR**TCLHPCRNT**	Y	✓	0
FLA_73_I2 / FRI_32_I2*	14/31	**CRPDQSSCRGHHSCCSGNCCYKFFSGTCRPNACGIIE**	Y	0	✓
FLA_93_M / FRI_53_M^	85/40	**CCSQDCWVCIPCCPNG**SP	Y	✓	0
FLA_117_O1 / FRI_74_O1*	4/17	**DCAGQGESCFTRPCCSGLQCTGPRPLGVCQ**	Y	0	✓
FLA_127_O1 / FRI_59_O1^	7076/1102	**DCGERGEGCYTRPCCPGLECVGGRPGGLCQY**	Y	✓	0
FLA_126_O1 / FRI_67_O1^*	93/44	DCGERGEGCYTRPCCPGLE**CRGTHAGGLCQY**	Y	✓	✓
FLA_133_O1 / FRI_62_O1*	330/154	**DCGNQGEGCRTRRCCPGLECIGGYPGGVCQW**	Y	0	✓
FLA_140_O1 / FRI_66_O1*	2962/76	**DCGNRGEGCYTRPCCPGLECRGTHAGGLCQW**	Y	0	✓
FLA_147_O1 / FRI_72_O1^	21/166	ECGEQGQGCYTRPCCPGQE**CVAGATGGGVCL**	Y	✓	0
FLA_153_O1 / FRI_78_O1^*	2009/31	**IPVGGFCALEGENCRIRPCCPDLSCSDNTGFECH**	Y	✓	✓
FLA_154_O1 / FRI_79_O1^*	781/27	**IPVGGFCALEGENCRIRPCCPGLSCSDNTGFECH**	Y	✓	✓
FLA_163_O2 / FRI_95_O2*	36/15	**KSTAQKWWDGECPGSSNGCRSPWDCCAGDCAEGYCVLR**	Y	0	✓
FLA_183_O3 / FRI_97_O3*	35/19	TTDEECEEYCEQR**NKDCCGLTNGEPR**CASMCF	Y	0	✓
FLA_185_O3 / FRI_101_O3^*	129/19	TTEECEEYCEQR**NKDCCGLTNGEPR**CASMCF	Y	✓	✓
FLA_196_T / FRI_104_T*	114/2	**GCCPDIAMCCVGI***	Y	0	✓
FLA_197_T / FRI_103_T^*	18/39	**SCCPDIAMCCVGI**	Y	✓	✓
FLA_201_V / FRI_108_V*	16/22	**DECVTCDGEECCGR**CTCVWGDCDCVDW	Y	0	✓
FRI_02_A	11	**DCCNLPACAASNPGLCT**	Y	0	✓
FRI_03_A^	3	**DCCNDPACTVNNPGLCT**	Y	✓	✓
FRI_04_A	87	**DCCSSAACAHHTPS**C	Y	0	✓
FRI_08_A	21	**GCCSDPPCAHHNPSC**	Y	0	✓
FRI_11_A	17	**GCCSNPPCAHHNPDC**	Y	0	✓
FRI_13_A	3	**GCCSDPPCAHHNPDC**	Y	0	✓
FRI_5_A	8	**GCCSNPPCAHHNPAC**	Y	0	✓
FRI_17_B1	5	AGHGGAATDVRSADNTLKRLLHDFRRALKRR**SDSRYDRKR** SISVNLDTEILAETVRGSQEDSREASVAGSLEMLEEAGR	N	0	✓
FRI_20_Conophy	17	STEQCWYCSFGKCVWPIVCCGSGGCQIGTAEANTCRPQAGERFPCKVIGKSCVMNNPSD**PSNYYGHCIGAEGHGVCCVDDNTCTAHSVCP**	Y	0	✓
FRI_23_Conophy	4	**STEQCWYCSFGKCVWPIVCCGSGGCQIGTPEANTCRPQAGQHFPCK**VIGK**SCVMNNPSDPSNYYGRCIGAEGHGICCVDDNTCTAHSVCP**	Y	0	✓
FRI_33_I2	40	CLSDGTVCRHHQQCCHRSCCFR**TCLHPCR**LGTRATFQEFILH	Y	0	✓
FRI_39_I2	4	CFPEGNRCRRNFHCCRENCCR**GFCQHPCGIA**	Y	0	✓
FRI_42_M	45	**YCPRPVC**	Y	0	✓
FRI_63_O1	145	DCGNQGEGCYTRPCCPGLE**CHGTHAGGLCQW**	Y	0	✓
FRI_68_O1	31	NCGNIGEGCYTR**RCCPGLECIGGYPGGV**CQW	Y	0	✓

FRI_76_O1^	5	**ACGKEGER**CLIRPCCTGLTCSGIIGGKCES	Y	✓	✓
FRI_77_O1	41	**IPVGGFCALEGQNCRIRPCCPGLSCSDNTGFECH**	Y	0	✓
FRI_80_O1	3	IPVGG**DCAGQGESCFKRPCCSGLQCTGPRPLGVCQ**	Y	0	✓
FRI_81_O1	3	LI**FGGWCGTEGESCKILPCCSDLTCSGIFEGICQS**	Y	0	✓
FRI_82_O1	2	**DGMESLKSVGLCWKEGLPCIIFPCCPGLTCGDGIIGDTCQS S**	Y	0	✓
FRI_104_T	2	**GCCPDIAMCCVGI**	Y	0	✓
FRI_106_V	3	R**DCTTCSGEECCGR**CTCTWGDNCDCIAW	Y	0	✓
